# Cation‐Engineered Gradient Interfacial Structure Toward Dendrite‐Free and Shuttle‐Free Aqueous Zn‐Iodine Batteries

**DOI:** 10.1002/advs.202509239

**Published:** 2025-07-28

**Authors:** Jiayi Li, Xiao Zhang, Xinming Xu, Bingqing Xie, Yuchun Wang, Long Su, Hansen Wang, Chuying Ouyang, Xinpei Gao

**Affiliations:** ^1^ School of Chemistry and Chemical Engineering Hainan University Haikou 570228 P. R. China; ^2^ Key Laboratory of Colloid and Interface Chemistry Shandong University, Ministry of Education Jinan 250100 P. R. China; ^3^ 21C Lab, Contemporary Amperex Technology Limited (CATL) Ningde 352100 P. R. China; ^4^ Department of Physics Jiangxi Normal University Nanchang 330022 P. R. China

**Keywords:** aqueous Zn‐iodine batteries, deposition behavior, electric double layer, gradient interfacial structure, ion desolvation

## Abstract

Uncontrolled dendrite growth, water‐induced side reactions, and polyiodide shuttling remain critical in aqueous Zn‐iodine batteries (AZIBs). Herein, an “electric double layer (EDL)‐directed regulator” strategy utilizing amphiphilic acetylcholine cation (ACh^+^) as interfacial modifiers is proposed. The directed adsorption of ACh^+^ on the Zn anode surface assembles a hydrophobic‐hydrophilic gradient interfacial structure. The hydrophobic inner layer establishes a water‐poor EDL structure, reducing direct Zn‐electrolyte contact and suppressing side reactions. Meanwhile, the hydrophilic outer layer disrupts the original H_2_O‐H_2_O within EDL structure, lowering water activity and reducing the Zn^2+^ desolvation energy barrier. When coupled with an I_2_ cathode, dissolvable polyiodide anions are captured by ACh^+^ via electrostatic interactions, effectively inhibiting the polyiodide shuttles. Consequently, the Zn anode with optimized EDL delivers a high Coulombic efficiency (CE) of 99.82%, with remarkable stability over 3700 h at 1.0 mA cm^−2^/1.0 mAh cm^−2^ and 1500 h at 10 mA cm^−2^/1.0 mAh cm^−2^. Moreover, the Zn‐I_2_ full cell exhibits an ultralow capacity decay rate of merely 0.000512% per cycle over 25000 cycles at 2.0 A g^−1^. This work provides an effective EDL regulation strategy for optimizing the Zn anode interfacial chemistry toward the advanced AZIBs.

## Graphical abstract

This paper proposes an “EDL‐directed regulator” strategy that constructs hydrophobic‐hydrophilic gradient interfaces to stabilize Zn anode by forming a water‐poor EDL and lowering the desolvation barriers Zn^2+^. Meanwhile, the ACh^+^‐containing electrolyte suppresses polyiodide shuttling via electrostatic capture, enhancing the long‐term reversibility and cycling performance of aqueous Zn‐iodine batteries (AZIBs).

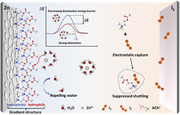

## 1 Introduction

Aqueous Zn‐iodine batteries (AZIBs) stand out due to their intrinsic safety, abundant natural resources (55 µg L^−1^ in seawater), relatively high specific capacity (211 mAh g^−1^ based on I^−^/I_2_ redox couple), and high discharge plateau (1.38 V vs Zn/Zn^2+^).^[^ ^1^ ^]^ However, their practical application remains hindered by uncontrolled dendrite growth, water‐induced side reactions, and the polyiodide shuttle effect.^[^ ^2^ ^]^ Specifically, the formation of Zn dendrites primarily stems from the inhomogeneous electric field distribution and the uncontrollable 2D diffusion of Zn^2+^, which ultimately tends to result in internal short circuits.^[^ ^3^ ^]^ Besides, the undesirable hydrogen evolution reaction (HER) and corrosion reactions occur within the electric double layer (EDL) on the Zn anode surface, thereby compromising the Coulombic efficiency (CE) and cycling stability of AZIBs.^[^ ^4^ ^]^ Moreover, the severe dissolution/diffusion of water‐soluble polyiodide (I_3_^−^, I_5_^−^ etc.) intermediates in electrolytes results in loss of active materials and Zn anode corrosion, leading to rapid capacity decay.

Actually, the Zn anode reversibility is fundamentally determined by the EDL structure where the Zn^2+^ plating/striping reaction occurs.^[^ ^5^ ^]^ The solvation structure of Zn^2+^ is dominated by the water molecules in conventional aqueous electrolytes. During the Zn deposition process, the desolvation of hydrated Zn^2+^ releases free water at the anode surface.^[^ ^6^ ^]^ As a result, the water‐dominant EDL structure accelerates side reactions, while destabilizing Zn^2+^ flux homogeneity, promoting dendritic growth.^[^ ^7^ ^]^ Hence, diverse strategies,^[^ ^8^ ^]^ including electrode coatings^[^ ^9^ ^]^ and electrolyte optimization (e.g., quasi‐solid electrolytes,^[^ ^10^ ^]^ high‐concentration electrolytes,^[^ ^11^ ^]^ and eutectic electrolytes^[^ ^12^ ^]^), have been devoted to recognizing the EDL structure and mitigating polyiodide shuttling in AZIBs. Particularly, electrolyte additive strategies have emerged as one of the most promising approaches for its simplicity and effectiveness.^[^ ^13^ ^]^ Certain molecules with specialized structures are designed as additives that can adsorb at the interface to remodel the EDL structure. In other words, the structure and properties of the additive directly determine the composition of EDL. While hydrophobic additives (e.g., SDBS,^[^ ^14^ ^]^ DDBA⁺, DTA⁺, TEMA⁺.^[^ ^15^ ^]^) minimize water content in EDL and guide Zn deposition by forming a hydrophobic protective layer, their high interfacial resistance increases Zn^2+^ desolvation energy, leading to sluggish kinetics. Conversely, hydrophilic additives (e.g., trehalose,^[^ ^16^ ^]^ maltitol,^[^ ^17^ ^]^ tripropylene glycol,^[^ ^18^ ^]^) facilitate ion desolvation but often fail to construct an effective hydrophobic barrier on the electrode surface, thereby exhibiting limited suppression of water‐induced side reactions. Considering that most additives can only unilaterally suppress HER or facilitate ion desolvation, how to address the intrinsic trade‐off between interfacial kinetics and side reaction inhibition ability is critical for developing high‐efficiency electrolyte additives.

**Figure 1:**
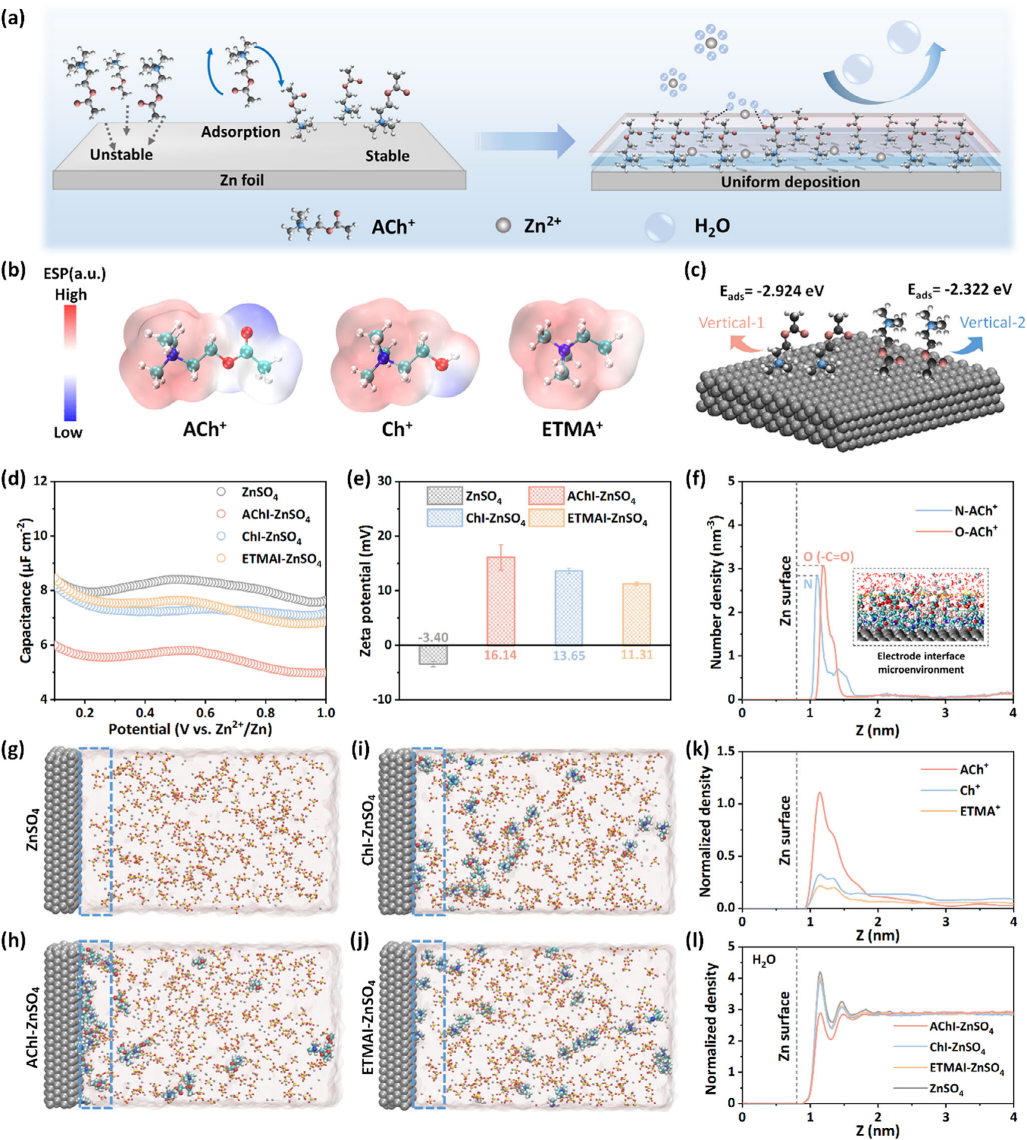
a) The schematic illustrations of adsorption progress and optimization mechanism of ACh^+^ on Zn surface. b) ESP mappings of ACh^+^, Ch^+^, and ETMA^+^. c) The schematic illustrations of ACh^+^ with different vertical adsorption orientations on Zn anode. d) Differential capacitance curves and e) Zeta potential of Zn metal in different electrolytes. The error bars represent the standard deviation from three independent measurements. f) Number density profiles of O(─C═O) and N atoms of ACh^+^. MD simulation snapshots of g) ZnSO_4_, h) AChI‐ZnSO_4_, i) ChI‐ZnSO_4_, and j) ETMAI‐ZnSO_4_ electrolytes. Normalized density profiles of k) ACh^+^, Ch^+^, ETMA^+^, and l) H_2_O within EDL in various electrolytes.

In this work, an “EDL‐directed regulator” strategy is proposed to construct gradient interfacial structures for stabilizing AZIBs. Acetylcholine iodide (AChI), choline iodide (ChI), and ethyltrimethylammonium iodide (ETMAI) (structural formula shown in Figure S1, Supporting Information) were chosen as EDL‐directed regulators to prove the effectiveness of this strategy. Among these cations, ACh^+^ exhibits the strongest adsorption affinity and leverages its asymmetric electrostatic potential distribution to directionally anchor onto the Zn surface, thereby constructing a hydrophobic‐hydrophilic gradient interfacial structure (**Figure** **1**a). The hydrophobic inner layer establishes a water‐poor EDL structure, reducing direct Zn‐electrolyte contact and suppressing side reactions. Moreover, the hydrophilic outer layer disrupts the original H_2_O‐H_2_O within EDL structure, lowering the free water activity and reducing the Zn^2+^ desolvation energy barrier. Simultaneously, the selective adsorption of ACh^+^ on different crystal planes facilitates the preferential deposition of Zn^2+^ along the Zn (002) plane, while its electrostatic shielding effect homogenizes the interfacial electric field, suppressing dendrite formation. The multifaceted role of ACh^+^ at the electrode‐electrolyte interface effectively inhibits HER and dendrite growth, ensuring highly reversible plating/stripping chemistry of the Zn anode. Consequently, the assembled Zn//Zn symmetrical cell demonstrates an ultra‐long lifespan of more than 3700 h at 1.0 mA cm^−2^/1.0 mAh cm^−2^ with a high CE of 99.82%. Fortunately, the strong electrostatic interaction between the quaternary ammonium cation and polyiodide anions effectively prevents shuttle effects, enabling the Zn‐I_2_ full battery with AChI to deliver highly reversible cycling with an ultralow capacity decay rate of only 0.000512% per cycle over 25 000 cycles at 2.0 A g^−1^. This EDL regulation strategy provides valuable insights into optimizing interfacial chemistry for advanced battery systems.

## 2 Results and Discussion

To validate the effectiveness of our “EDL‐directed regulator” strategy in regulating the interfacial behavior of the AZIBs, three ionic liquids with the same anions (I^−^) but series quaternary ammonium cations (ACh^+^, Ch^+^, and ETMA^+^) were selected as regulators. Specifically, ETMA^+^ serves as a typically hydrophobic cation, whereas ACh^+^ and Ch^+^ demonstrate hydrophobic‐hydrophilic structural characteristics. To elucidate the essence of the amphipathic characteristic in determining the cation properties, the electrostatic potential (ESP) mappings were calculated for the three cations. As shown in Figure 1b, the introduction of hydrophilic groups (─C═O and ─OH) in ACh^+^ and Ch^+^ enhances the asymmetry in electrostatic potential distribution across their molecular frameworks. Comparative analysis with the ESP profile of H_2_O (Figure S2, Supporting Information) suggests that both ACh^+^ and Ch^+^ cations are anticipated to exhibit amphiphilic characteristics in aqueous solution: the ─N^+^(CH_3_)_3_ terminus remains hydrophobic, while the polar ─C═O and ─OH groups confer hydrophilicity. Furthermore, the density functional theory (DFT) calculation was performed to illustrate the influence of structural differences on the interfacial adsorption behavior of quaternary ammonium cations. Figure 1c display two vertical adsorption configurations and the corresponding adsorption energies of three cations on the Zn surface (Figure S3, Supporting Information). In the Vertical‐1 configuration, the ─N^+^(CH_3_)_3_ group is oriented downward, whereas Vertical‐2 corresponds to an upward orientation of the ─N^+^(CH_3_)_3_ moiety. The adsorption energies for Vertical‐1 configuration (ACh^+^: −2.924 eV, Ch^+^: −2.852 eV, ETMA^+^: −2.737 eV) are higher than those in the Vertical‐2 configuration (ACh^+^: −2.322 eV, Ch^+^: −2.245 eV, ETMA^+^: −2.130 eV), indicating a preference for downward adsorption of the ─N^+^(CH_3_)_3_ moiety in vertical orientations. Although horizontal adsorption configurations exhibit slightly higher adsorption energy (Figure S4, Supporting Information), their larger footprint limits surface coverage, whereas the vertical adsorption configurations minimize area occupation via spatial efficiency, rendering them the preferred mode for enhanced interfacial capacity. As shown in Figure S5 (Supporting Information), when ACh^+^ accumulates at the interface, it adopts a vertically aligned adsorption configuration with higher overall adsorption energy. This behavior is further corroborated by MD simulations (Figure S6, Supporting Information).

To compare the effect of quaternary ammonium cations on electrochemical performance, 8 mM regulators (details can be found in Figure S7, Supporting Information) were introduced into 2 M ZnSO_4_ aqueous solution to form electrolytes with trace amount additives. For clarity, the obtained electrolytes are denoted as AChI‐ZnSO_4_, ChI‐ZnSO_4_, and ETMAI‐ZnSO_4_, respectively. Interestingly, while all three regulators improve the cycling performance, the AChI‐ZnSO_4_ electrolyte outperforms the others in both CE (Figure S8, Supporting Information) and cycle life (Figure S9, Supporting Information). Compared to the baseline 2 M ZnSO_4_ electrolyte, the regulators exhibit minimal influence on bulk phase properties (Figure S10, Supporting Information), with performance enhancement originating predominantly from its regulation of Zn deposition behavior. Given the pivotal role of EDL structure in governing electrochemical performance, comprehensive characterizations were employed to elucidate the interfacial adsorption behaviors of quaternary ammonium cations.

Differential capacitance (DC) tests (Figure 1d) demonstrate reduced capacitance for AChI‐ZnSO_4_, ChI‐ZnSO_4_, and ETMAI‐ZnSO_4_ electrolytes compared to the ZnSO_4_ electrolyte, primarily due to the increased thickness of the EDL caused by the incorporation of bulky cations.^[^ ^19^ ^]^ Consistent with experimental observation, the adsorption energies of ACh^+^, Ch^+^, and ETMA^+^ on Zn surfaces are higher than that of H_2_O (−0.482 eV) (Figure S3, Supporting Information), suggesting preferential adsorption of these cations onto the Zn anode surface. Simultaneously, the zeta potential analysis reveals a surface charge reverses from −3.40 mV in 2 M ZnSO_4_ electrolyte to 16.14, 13.65, and 11.31 mV in AChI‐ZnSO_4_, ChI‐ZnSO_4_, and ETMAI‐ZnSO_4_ electrolytes, respectively (Figure 1e), with the largest shift in AChI‐ZnSO_4_ electrolyte aligning with its superior adsorption capacity.^[^ ^20^ ^]^ Differential charge density analysis of the optimized adsorption configuration reveals pronounced orbital overlap between quaternary ammonium cations and the Zn anode, highlighting robust interfacial coupling and facilitated charge transfer (Figure S11, Supporting Information). This competitive process successfully displaces water content within the EDL structure. The density distribution profiles illustrate the spatial arrangement of interfacial ions and H_2_O molecules within the EDL relative to the distance from the Zn surface.^[^ ^21^ ^]^ As shown in Figure 1f, the distance between the Zn surface and the N atoms in ACh^+^ is shorter than that of Zn between O atoms, demonstrating a vertically oriented adsorption configuration of ACh^+^. This specific molecular arrangement establishes a hydrophobic‐hydrophilic gradient EDL structure, where hydrophilic moieties extend outward from the Zn surface while hydrophobic groups remain anchored at the interface.

Molecular dynamics (MD) simulations were carried out to further elucidate the adsorption behavior of the three cations on the Zn surface. Simulated snapshots of the ZnSO_4_ electrolyte, AChI‐ZnSO_4_, ChI‐ZnSO_4_, and ETMAI‐ZnSO_4_ electrolytes are presented in Figure 1g–j. Under identical conditions, all three cations appear in the EDL structure. Specifically, ACh^+^ exhibits stronger adsorption on the Zn surface compared to Ch^+^ and ETMA^+^, which leads to a localized enrichment of ACh^+^ at the interface to form a water‐poor EDL. X‐ray Photoemission Spectroscopy (XPS) results further confirm the interaction. The N 1s spectra (Figure S12, Supporting Information) reveal the distinct N‐Zn signals on Zn surfaces immersed in electrolytes containing AChI, ChI, and ETMAI, whereas they are absent in the ZnSO_4_ electrolyte. Additionally, energy dispersive spectroscopy (EDS) mapping of the Zn metal surface after immersion (Figure S13, Supporting Information) reveals a uniform distribution of Zn, C, N, and O elements, signifying homogeneous coverage of the Zn anode by quaternary ammonium cations. This coverage enhances interfacial transfer resistance (Figure S14, Supporting Information) and reduces the contact angle (Figure S15, Supporting Information).^[^ ^22^ ^]^ Additive‐containing electrolytes exhibit a lower contact angle than the ZnSO_4_ electrolyte (Figure S16, Supporting Information), indicating enhanced surface affinity and wettability toward the Zn surface. In addition, the normalized density distribution (Figure 1k) confirms the adsorption of all three cations on the Zn surface, reducing the interfacial water content. Notably, ACh^+^ demonstrates the strongest interfacial affinity, leading to the greatest interfacial water reduction as evidenced in Figure 1l. This successfully prevents direct contact between H_2_O and the Zn anode.^[^ ^23^ ^]^ Overall, the adsorption of quaternary ammonium cations, acting as EDL‐directing regulators, induces a transition of the EDL from a water‐rich to a water‐poor structure (Figure S17, Supporting Information), which is expected to stabilize the Zn anode and inhibit side reactions.

**Figure 2:**
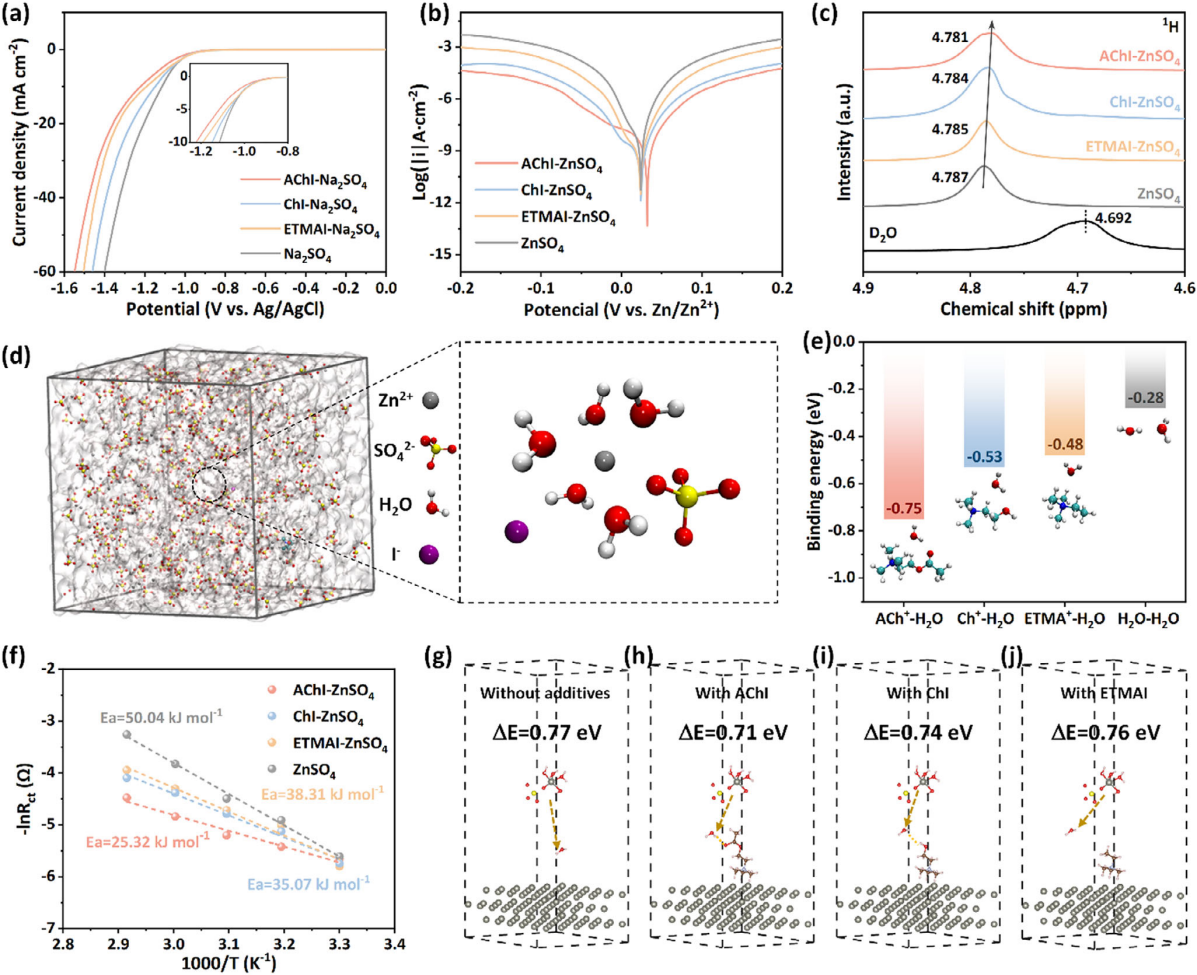
a) LSV (scan rate of 1.0 mV s^−1^) and b) Tafel (scan rate of 10 mV s^−1^) measurement of different electrolytes. c) ^1^H NMR spectra of different electrolytes. d) Representative simulation snapshot and solvation structure for AChI‐ZnSO_4_ electrolyte. e) Binding energy of ACh^+^, Ch^+^, ETMA^+^, and H_2_O with H_2_O. f) Arrhenius curves of activation energies with different electrolytes. g–j) Desolvation energy barrier for the removal of one water molecule from [Zn(H_2_O)_5_SO_4_] at the Zn surface with/without quaternary ammonium cations adsorption.

To accurately measure the suppression of the side reaction, linear sweep voltammetry (LSV) was employed to evaluate the HER potentials of different electrolytes.^[^ ^24^ ^]^ To eliminate the influence of Zn^2+^ during electrochemical measurements, all tests were conducted in Na_2_SO_4_ electrolyte. **Figure** **2**a shows that the AChI‐containing electrolyte exhibits a lower HER potential than those with ChI, ETMAI, and the baseline Na_2_SO_4_ electrolyte, confirming that the HER can be suppressed by the AChI‐induced water‐poor EDL. Subsequently, a linear polarization (Tafel) experiment analyzed the corrosion protection effects of various additives on Zn anodes.^[^ ^25^ ^]^ The results show that the corrosion current densities in additive‐containing electrolytes are lower than those of pure ZnSO_4_, with the AChI‐ZnSO_4_ electrolyte exhibiting the lowest corrosion current density (Figure 2b). Notably, the suppression of side reactions primarily arises from the formation of a water‐poor EDL structure, rather than from a suppression in bulk water activity. ^1^H NMR (Figure 2c) identifies the enhanced Zn^2+^‐H_2_O interaction, with the proton resonance shifting from 4.692 to 4.787 ppm after the addition of ZnSO_4_, indicating reduced electron shielding of water protons in H_2_O.^[^ ^26^ ^]^ Interestingly, upon the introduction of additives, the proton chemical shift exhibits minimal variation, suggesting a negligible impact on the water coordination environment. The local environment of water molecules in Zn^2+^ solvation structure was explored through MD simulations. The radial distribution function (RDF) and average coordination number (ACN) confirm the dominance of [Zn(H_2_O)_5_(SO_4_)] solvation structures in baseline ZnSO_4_ electrolyte (Figure S18, Supporting Information). Expectable, although Zn^2+^ exhibits strong interactions with I^−^ (Figure S19, Supporting Information), the Zn^2+^ solvation structure remains unchanged with the addition of additive (Figure 2d; Figure S20, Supporting Information). Consistently, Fourier transform infrared spectroscopy (FTIR) detects negligible variation in O─H stretching vibration across all electrolytes (Figure S21, Supporting Information). Thus, the introduction of low‐concentration additives exerts a negligible effect on the local environment of water molecules in bulk electrolytes.

Nevertheless, the orientated adsorption of quaternary ammonium cations alters the water molecule activity within the EDL structures. As shown in Figure 2e, the binding energies of ACh^+^, Ch^+^, and ETMA^+^ with H_2_O are −0.75, −0.53, and −0.48 eV, respectively, which are higher than the original H_2_O‐H_2_O hydrogen bond. This suggests that the ─C═O group in ACh^+^ and the ─OH group in Ch^+^ may disrupt hydrogen bonds between H_2_O, thereby reducing their activity. In addition, ^1^H NMR characterization was performed for the electrolyte with increased additive concentrations (from 8 mM to 80 mM) to further confirm the ACh^+^‐water hydrogen bond interactions, while the electrolyte with same concentration of KI additive as the control system. As shown in Figure S22 (Supporting Information), there are no obvious ^1^H chemical shifts upon KI addition. In contrast, the AChI‐ZnSO_4_ electrolyte exhibits an obvious upfield shift (4.771 ppm) in proton resonance, confirming the formation of the hydrogen bond between ACh^+^ and interfacial H_2_O.^[^ ^27^ ^]^ Thus, there exists a hydrophilic layer in EDL that is expected to confine H_2_O molecules and facilitate ion desolvation. To confirm this, activation energies (E_a_) were calculated using the Arrhenius equation (Equation S2, Supporting Information) to evaluate the desolvation process.^[^ ^28^ ^]^ Activation energies were derived from Nyquist plots recorded at various temperatures (30–70 °C) (Figure S23, Supporting Information). The AChI‐ZnSO_4_ demonstrates decreased E_a_ values (25.32 kJ mol^−1^) compared with the ZnSO_4_ electrolyte (50.04 kJ mol^−1^), outperforming ChI‐ZnSO_4_ (35.07 kJ mol^−1^) and ETMAI‐ZnSO_4_ (38.31 kJ mol^−1^) electrolytes (Figure 2f). Furthermore, DFT calculations reveal the energy desolvation barrier (ΔE) decreases from 0.77 eV in ZnSO_4_ electrolyte without cation adsorption to 0.71, 0.74, and 0.76 eV for AChI‐ZnSO_4_, ChI‐ZnSO_4_, and ETMAI‐ZnSO_4_ electrolytes, respectively (Figure 2g–j). Notably, ACh^+^, with its ─C═O functional group, reduces the desolvation energy barrier of hydrated Zn^2+^, aligning with its stronger binding energy with Zn^2+^ (Figure S24, Supporting Information).^[^ ^29^ ^]^ By comparison, the weakly polar Ch^+^ and ETMA^+^ exhibit minimal effects. Overall, although trace additives have negligible influence on the bulk solvation structure of Zn^2+^, their interfacial adsorption reshapes the EDL into a functional hydrophobic‐hydrophilic interfacial structure, where the hydrophobic near‐anode regions minimize contact with H_2_O and inhibit water‐induced side reactions, while hydrophilic zones enhance desolvation efficiency.

**Figure 3:**
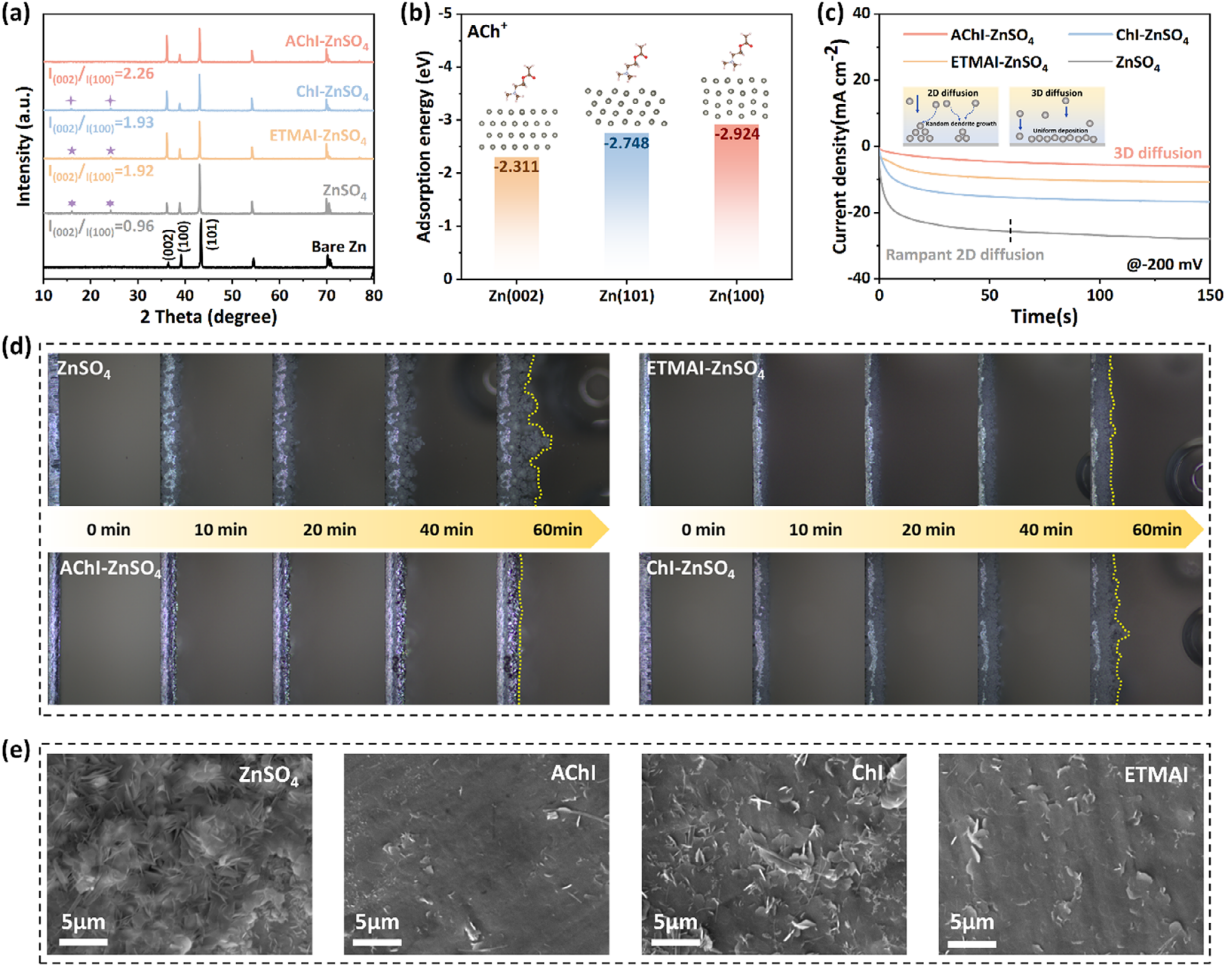
a) XRD patterns of Zn anodes after 50 cycles at 2.0 mA cm^−2^ and 1.0 mAh cm^−2^. b) Adsorption energy of ACh^+^ on different crystal planes of Zn anode. c) CA curves of Zn anodes in different electrolytes at an overpotential of −200 mV. d) In situ optical microscopy images of the Zn plating process in different electrolytes at the current density of 5.0 mA cm^−2^. e) SEM images of Zn anodes in different electrolytes after 50 cycles at 1.0 mA cm^−2^ and 1.0 mAh cm^−2^.

In addition to suppressing side reactions and facilitating ion desolvation, the introduction of EDL‐directed regulator also plays a key role in regulating the deposition behavior of Zn^2+^. The Zn anodes after 50 cycles in Zn//Zn cells were characterized by X‐ray diffraction (XRD). Notably, the by‐product peak is reduced upon the introduction of ChI and ETMAI, and is almost eliminated in the AChI‐ZnSO_4_ electrolyte (**Figure** **3**a), again confirming the inhibition of corrosion by EDL‐directed regulator. Moreover, DFT calculations were performed to investigate the adsorption energies of three quaternary ammonium cations (ACh^+^, Ch^+^, and ETMA^+^), H_2_O, and Zn^2+^ on different Zn crystal planes. As shown in Figure 3b and Figure S25 (Supporting Information), all three cations exhibit lower adsorption energies than H_2_O and Zn^2+^, indicating a stronger affinity toward the Zn surface. This preferential adsorption facilitates the formation of a positively charged electrostatic shielding layer, which mitigates localized Zn^2+^ accumulation via electrostatic repulsion. Compared with Zn (002) plane (−2.311 eV), ACh^+^ exhibits stronger adsorption on the Zn (100) (−2.924 eV) and Zn (101) (−2.748 eV) planes, and thus preferentially adsorbs on (100) plane, then on (101) plane, leaving the (002) plane more exposed. This tendency is supported by the increased (002)/(100) peak intensity ratio observed in XRD patterns. Ch^+^ and ETMA^+^ show comparable adsorption behavior. The increased exposure of Zn (002) planes effectively inhibits dendrite growth from a kinetic perspective, while simultaneously offering the most thermodynamically stable configuration due to their lowest surface energy, thereby reducing parasitic side reactions. Furthermore, the Zn^2+^ nucleation behaviors were evaluated through chronoamperometry (CA) in Zn//Zn cells at a fixed overpotential of −200 mV (Figure 3c). In the ZnSO_4_ electrolyte, the current density at the Zn anode continuously increases, indicating an uncontrolled 2D diffusion process and a tendency for Zn^2+^ to deposit on preformed tips, promoting the growth of Zn dendrites. By comparison, with the introduction of additives, particularly AChI‐ZnSO_4_, Zn^2+^ exhibits a stronger tendency toward a 3D diffusion process.^[^ ^30^ ^]^

Atomic force microscopy (AFM) characterization demonstrates superior dendrite suppression in AChI‐ZnSO_4_ electrolytes, remaining relatively smooth without obvious dendrite growth versus pronounced dendritic features in ZnSO_4_ electrolytes (Figure S26, Supporting Information). In situ optical microscopy further monitored the evolution of zinc electrodeposition in different electrolytes. In the ZnSO_4,_ ChI‐ZnSO_4_, and ETMAI‐ZnSO_4_ electrolytes, noticeable bubbles and loose Zn dendrites appear within 40 min, while the Zn deposition morphology remains uniform and flat, with no bubble formation in AChI‐ZnSO_4_ and electrolyte (Figure 3d). Scanning electron microscopy (SEM) analysis confirms this trend, with the Zn anode after 50^th^ cycle remaining flat without dendrite formation in additive‐containing electrolytes, while the loose structure with severe dendritic growth is detected on Zn anode cycled in ZnSO_4_ electrolyte (Figure 3e). These observations suggest that the adsorption‐driven hydrophobic‐hydrophilic gradient interfacial structure of ACh^+^ cations precisely guides uniform Zn^2+^ deposition, while concurrently inhibiting water decomposition, protecting the anode from corrosion, and minimizing dendrite growth and side reactions.

**Figure 4:**
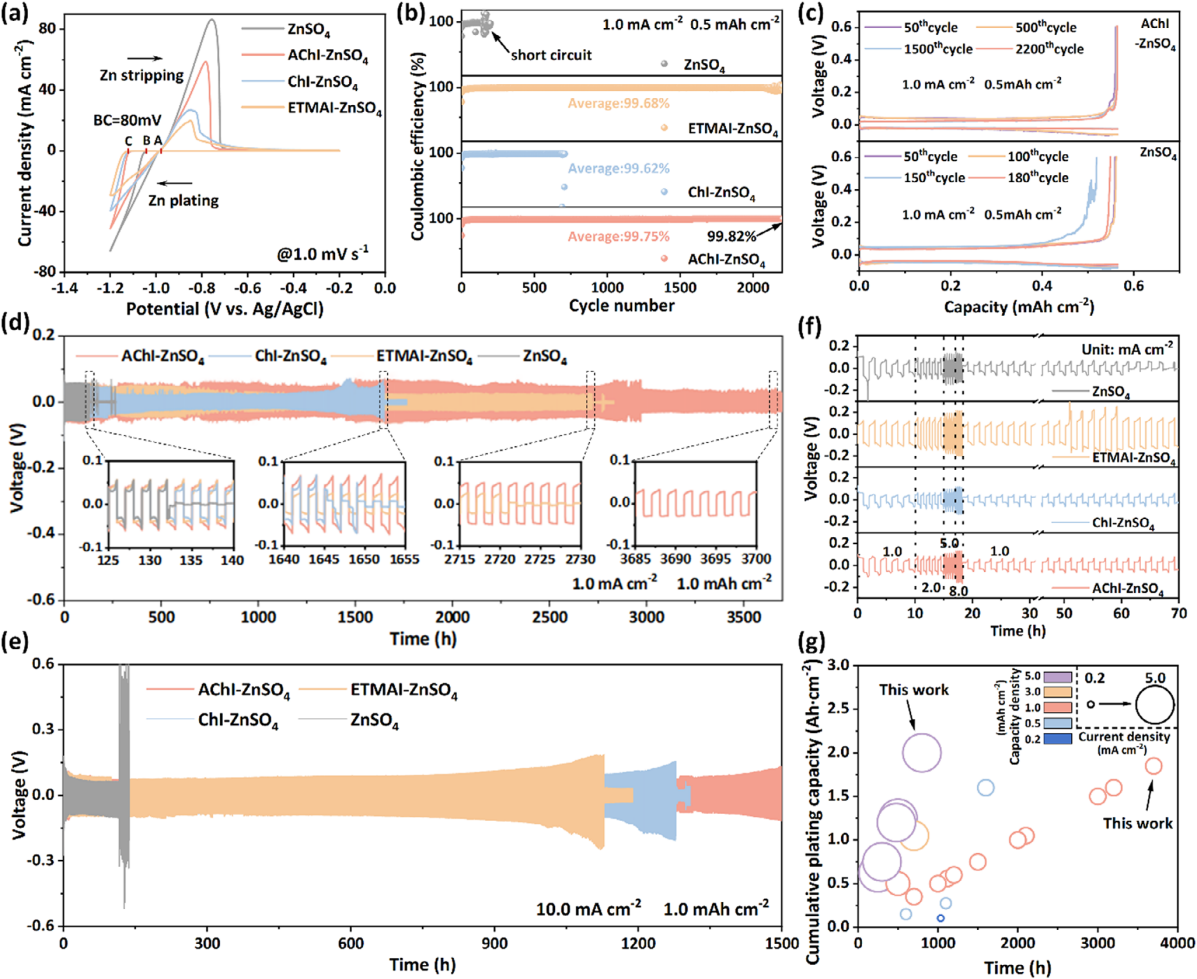
a) CV profiles of Zn plating/stripping with Ti foil as working, Zn as counter, and Ag/AgCl as reference electrode at 1.0 mV s^−1^ in different electrolytes. b) CE performances of Zn//Cu asymmetric cells with different electrolytes at 1.0 mA cm^−2^ and 0.5 mAh cm^−2^. c) Voltage profiles of Zn//Cu asymmetric cells at various cycles in AChI‐ZnSO_4_ and ZnSO_4_ electrolytes. Long‐term cycling performances of Zn//Zn symmetrical cells at d) 1.0 mA cm^−2^/1.0 mAh cm^−2^ and e) 10.0 mA cm^−2^/1.0 mAh cm^−2^ using different electrolytes. f) Rate performances of Zn//Zn symmetrical cells with different electrolytes. g) Comparison of cumulative galvanic capacity and lifespan between this work and recently reported works using other electrolyte additives (Details can be found in Table S2, Supporting Information).

The role of the EDL‐directed regulator on the reversibility and cycling stability of the Zn anode is further evaluated. As shown in **Figure** **4**a, the CV profiles of the AChI‐ZnSO_4_ electrolyte within the potential range of −1.0–−0.2 V display a single peak, indicating the absence of electrochemical by‐product formation during cycling.^[^ ^31^ ^]^ Moreover, the peak current density of the CV curves remains stable over 10 cycles (Figure S27, Supporting Information), highlighting the improved reversibility and cycling stability imparted by AChI additive.^[^ ^24^ ^]^ Notably, the nucleation overpotential increases with the addition of EDL regulators, suggesting that the quaternary ammonium‐modified EDL reduces the nucleation radius, promoting dense Zn deposition.^[^ ^1^, ^32^ ^]^ This interfacial optimization is directly reflected in CE measurement using Zn//Cu asymmetric cells at 1.0 mA cm^−2^/0.5 mAh cm^−2^ (Figure 4b). The cell with ZnSO_4_ electrolyte suffers from rapid performance degradation, exhibiting CE fluctuations followed by cell failure after 120 cycles, which can be attributed to the severe Zn dendrite growth and side reactions. In contrast, the AChI‐ZnSO_4_ electrolyte achieves exceptional cycling stability, maintaining a CE of 99.82% over 2200 cycles with stable voltage profiles (Figure 4c; Figure S28, Supporting Information). Given the pivotal role of Zn anodes in enabling practical application, their reversibility was further evaluated through deep‐discharge tests (50% DOD_Zn_) in Zn//Cu half cells under a “preactivated mode” (Figure S29, Supporting Information). As shown in Figure S30 (Supporting Information), the AChI‐containing electrolyte enables stable cycling for over 300 h with an average CE of 99.34%, while the blank ZnSO_4_ cell fails after only 28 h (CE = 94.33%), confirming the additive's effectiveness in improving Zn reversibility under high DOD conditions.

The Zn//Zn symmetrical cells were further assembled to investigate the long‐term cycling performances and rate performances of different electrolytes. As shown in Figure 4f, the dual advantages of the reorganized EDL in suppressing side reactions and dendritic growth encourage the rate capability of electrolytes in the range of currents from 1.0 to 8.0 mA cm^−2^. Furthermore, the galvanostatic cycling of Zn//Zn symmetric cell with ZnSO_4_ experiences a short circuit after only 130 h at 1.0 mA cm^−2^/1.0 mAh cm^−2^, primarily due to dendrite growth leading to battery failure. In contrast, the lifespans of the ChI‐ZnSO_4_ and ETMAI‐ZnSO_4_ electrolytes are extended to 1645 and 2720 h, respectively, while the AChI‐ZnSO_4_ system achieves an impressive stable cycle of 3700 h (Figure 4d). Even at a higher current density of 10.0 mA cm^−2^ (Figure 4e), the AChI‐ZnSO_4_ electrolyte demonstrates a cycle life exceeding 1500 h, outperforming the ChI‐ZnSO_4_ (1100 h) and ETMAI‐ZnSO_4_ (1300 h) electrolytes. This improved performance is attributed to the reorganization of the EDL structure, which effectively inhibits dendrite growth and side reactions. However, differences in the functional groups of the cations affect their adsorption behavior on the Zn anode surface, resulting in variations in CE and cycle life across the electrolyte systems. Elevating the concentrations of ChI and ETMAI enhances their cycling performance (Figure S31, Supporting Information). This suggests that while all three additives improve battery performance, AChI achieves superior results at lower dosages due to its strongest adsorption affinity toward the Zn surface, which drives denser interfacial coverage and the spontaneous formation of a hydrophilic‐hydrophobic gradient, thereby ensuring both high efficacy and cost‐effectiveness. Notably, the cumulative galvanic capacity and cycle stability of the half‐cells assembled with AChI‐ZnSO_4_ demonstrate advantages over those reported in previous studies on electrolyte engineering strategy (Figure 4g).

**Figure 5:**
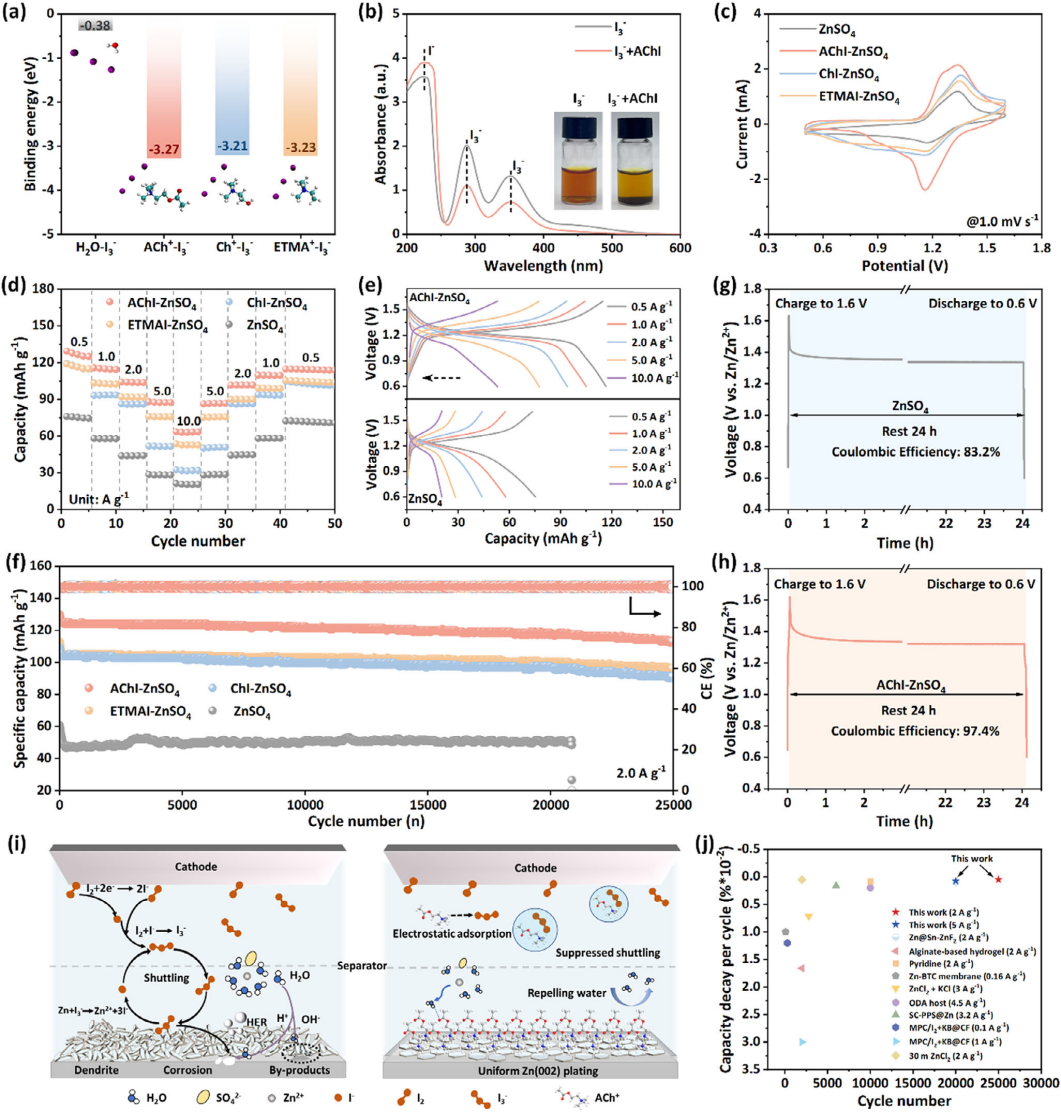
a) Binding energy of H_2_O, ACh^+^, Ch^+^, and ETMA^+^ with I_3_^−^. b) UV–vis spectra of polyiodide solutions with/without AChI. (The I_3_^−^ solution was prepared by dissolving I_2_ and KI in a 1:1 molar ratio in deionized water to achieve a concentration of 5 mM KI_3_.) c) CV profiles at 1.0 mV s^−1^ of Zn//I_2_ full cells with different electrolytes. d) Rate performances of Zn//I_2_ full cells with different electrolytes. e) Charge–discharge curves of AChI‐ZnSO_4_ and ZnSO_4_ electrolytes at various rates. f) Long‐term cycling performances of Zn//I_2_ full cells with different electrolytes at 2.0 A g^−1^. Self‐discharge test of Zn//I_2_ full cells using g) ZnSO_4_ and h) AChI‐ZnSO_4_ electrolytes. i) Schematics of optimization strategy without (left)/with (right) AChI. j) Comparison of the cycling performances between AChI‐optimized Zn‐I_2_ batteries and those reported in recent literature (Details can be found in Table S4, Supporting Information).

In addition to the challenges at the anode, the dissolution of iodine at the cathode and the shuttle effect of polyiodide present major hurdles for AZIBs. To address these limitations, the iodine cathode stabilization mechanisms enabled by three quaternary ammonium additives were systematically investigated. As shown in **Figure** **5**a, DFT calculations reveal stronger interactions between I_3_^−^ with ACh^+^ (−3.27 eV), Ch^+^(−3.21 eV), and ETMA^+^ (−3.23 eV) compared with H_2_O (−0.28 eV). This suggests that I_3_− is more likely to form complexes with ACh^+^, Ch^+^, and ETMA^+^ rather than dissolve in water.^[^ ^2e^ ^]^ Similar trends are observed for I^−^ and I_5_^−^ (Figure S32, Supporting Information). Experimental validation through polyiodide solution tests confirms this phenomenon. AChI, ChI, and ETMAI were added to the polyiodide solution, and the resulting digital photos were observed in Figure 5b and Figure S33 (Supporting Information). The introduction of AChI, ChI, and ETMAI additive induces a visible color transition from reddish brown to pale yellow alongside precipitate formation, accompanied by a decrease in the UV–vis absorption peaks of I_3_^−^ at 288 and 350 nm, directly correlating the decrease in polyiodide concentration. Both theoretical calculations and experimental results indicate that the quaternary ammonium cation effectively inhibits the shuttle of polyiodides, potentially enhancing the reversibility and stability of AZIBs.

Consequently, the electrochemical performance of Zn//I_2_ full cells was systematically evaluated through comprehensive electrochemical testing. Figure 5c shows an increased peak current in the CV curves of cells with mixed electrolytes compared to those using ZnSO_4_ alone, indicating enhanced long‐term reversibility in full cells with additives.^[^ ^1^, ^33^ ^]^ The rate performance of Zn//I_2_ full cells at various current densities from 0.5 to 10.0 mA cm^−2^ confirms the additive‐induced improvement in interfacial kinetic performance (Figure 5d). Notably, at higher current densities, the AChI‐ZnSO_4_ electrolyte delivers lower capacity. When the current density is reduced back to 0.5 mA cm^−2^, the AChI‐ZnSO_4_ electrolyte demonstrates 92% capacity retention, highlighting its enhanced rate capability. This performance trend is consistently reflected in the well‐preserved charge–discharge voltage profiles (Figure 5e; Figure S34, Supporting Information). The improved performance originates from reduced interfacial charge‐transfer resistance in AChI‐containing electrolytes, as confirmed by electrochemical analysis (Figure S35, Supporting Information).^[^ ^34^ ^]^ The long‐term cycle performance of the full cells is evaluated in Figure 5f. After 25000 cycles, Zn//I_2_ full cells employing AChI‐ZnSO_4_ (115.3 mAh g^−1^, 87.2% retention), ChI‐ZnSO_4_ (91.5 mAh g^−1^, 83.8%), and ETMAI‐ZnSO_4_ (96.5 mAh g^−1^, 85.4%) electrolytes demonstrate improved electrochemical performance than the baseline ZnSO_4_ electrolyte (48.41 mAh g^−1^, 61.3%). The same trend persists under a higher current density of 5.0 A g^−1^ (Figure S36, Supporting Information). Notably, even under a higher iodine loading of 2.5 mg cm^−2^, the AChI‐ZnSO_4_ electrolyte exhibits enhanced specific capacity and cycling stability, maintaining 93.2% of its initial capacity after 2000 cycles (Figure S37, Supporting Information). Moreover, the addition of AChI, ChI, and ETMAI to the ZnSO_4_ electrolyte suppresses the self‐discharge behavior of Zn//I_2_ full cells. After 24 h of resting, cells with AChI‐ZnSO_4_, ChI‐ZnSO_4_, and ETMAI‐ZnSO_4_ electrolytes exhibit high capacity retention of 97.4, 96.0, and 93.8%, respectively, outperforming the 83.2% retention observed for the ZnSO_4_ electrolyte (Figure 5g,h; Figure S38, Supporting Information). As shown in Figure S39 (Supporting Information), the Zn//I_2_ full cell assembled with the AChI‐ZnSO_4_ electrolyte successfully powers a hygrometer, demonstrating its practical applicability.

Overall, the degradation mechanisms in aqueous ZnSO_4_ electrolytes originate from water‐induced side reactions at the Zn anode interface. As illustrated in Figure 5i, proton generation through water activation initiates dual detrimental processes: H^+^ induces anode corrosion, and OH^−^ reacts with Zn^2+^ and SO_4_^2−^ to form insoluble zinc sulfate salts. Then, surface inhomogeneities from corrosion byproducts disrupt the electric field, promoting Zn^2+^ accumulation and dendrite growth. In addition, Lewis acid‐base interactions between iodide species generate soluble polyiodides (I_3_^−^/I_5_^−^), exacerbating capacity loss through redox shuttle effects. Encouragingly, upon AChI addition, ACh^+^ directionally adsorbs onto the anode surface, forming a hydrophobic‐hydrophilic gradient interfacial structure to promote Zn^2+^ desolvation and inhibit side reactions. On the cathode, the electrostatic interaction between ACh^+^ and I^−^ prevents polyiodide formation and shuttling, enabling long‐term reversibility and stability in Zn‐I_2_ batteries. As a result, the obtained AChI‐ZnSO_4_ electrolyte achieves an ultralow capacity decay rate of 0.000512% per cycle that surpasses recent studies for Zn‐I_2_ batteries (Figure 5j).

## 3 Conclusion

In summary, an “EDL‐directed regulator” strategy is proposed to construct gradient interfacial structures for stabling AZIBs is developed. Both experimental and calculation results highlight the crucial role of hydrophobic‐hydrophilic gradient interfacial structure in enhancing the reversibility of Zn anode. The hydrophobic inner layer establishes a water‐poor EDL structure, reducing direct Zn‐electrolyte contact and suppressing side reactions. Furthermore, the hydrophilic outer layer disrupts the original H_2_O‐H_2_O with EDL structure, lowering the free water activity and reducing the Zn^2+^ desolvation energy barrier. Moreover, the selective adsorption of ACh^+^ on different crystal planes facilitates the preferential deposition of Zn^2+^ along the Zn (002) plane, effectively suppressing dendrite formation. As a result, the electrolyte with the EDL‐directed regulator additive (AChI) enables the Zn anode to stably cycle for 3700 h at 1.0 mA cm^−2^/1.0 mAh cm^−2^, and over 1500 h at a high‐current density of 10 mA cm^−2^. The Zn//Cu cells sustain over 2000 cycles with an average CE of 99.82%. Furthermore, the assembled Zn//I_2_ full cell demonstrates an ultra‐long lifespan exceeding 25 000 cycles, with an exceptionally low capacity decay rate of 0.000512% per cycle, attributed to the electrostatic interaction between polyiodides and ACh^+^, which suppresses the shuttle effect. This work is expected to provide a reliable electrolyte additive strategy for regulating the Zn anode interfacial chemistry toward the advanced Zn metal batteries.

## Conflict of Interest

The authors declare no conflict of interest.

## Supporting information



Supporting Information

## Data Availability

The data that support the findings of this study are available in the supplementary material of this article.
