# Supplementary material for: Cation‐Engineered Gradient Interfacial Structure Toward Dendrite‐Free and Shuttle‐Free Aqueous Zn‐Iodine Batteries
Source: Adv Sci (Weinh). 2025 Jul 28;12(40):e09239. doi: 10.1002/advs.202509239 (PMC12561259; doi:10.1002/advs.202509239)
Supplement: Supplementary file 1 — Supporting Information [file ADVS-12-e09239-s001.docx]

Supporting Information

**Cation-Engineered Gradient Interfacial Structure toward Dendrite-Free and Shuttle-Free Aqueous Zn-Iodine Batteries**

*Jiayi Li, Xiao Zhang, Xinming Xu, Bingqing Xie, Yuchun Wang, Long Su*, Hansen Wang*, Chuying Ouyang, Xinpei Gao**

J. Li, B. Xie, L. Su, X. Gao

School of Chemistry and Chemical Engineering, Hainan University, Haikou 570228, P. R. China.

E-mail: [xpgao@hainanu.edu.cn](mailto:xpgao@hainanu.edu.cn); [longsu@hainanu.edu.cn](mailto:longsu@hainanu.edu.cn)

X. Zhang, X. Xu

Key Laboratory of Colloid and Interface Chemistry, Shandong University, Ministry of Education, Jinan, 250100, P. R. China.

Y. Wang, H. Wang, C. Ouyang

21C Lab, Contemporary Amperex Technology Limited (CATL), Ningde 352100, P. R. China.

E-mail: [WangHS@catl-21c.com](mailto:WangHS@catl-21c.com)

C. Ouyang

Department of Physics, Jiangxi Normal University, Nanchang 330022, P. R. China.

**Experimental Section**

**Materials:** ZnSO_4_·7H_2_O (AR, 99.0%), Na_2_SO_4_ (AR, 99.0%), acetylcholine iodide (AChI, ≥98.0%), choline iodide (ChI, ≥98.0%) and ethyltrimethylammonium iodide (ETMAI, ≥98.0%) were purchased from Aladdin Chemicals. Zn foil and Cu foil were purchased from Guangdong Canrd New Energy Technology Co., Ltd. 1-methyl-2-pyrrolidone (NMP) was purchased from J&K. Polyvinylidene fluoride (PVDF) was purchased from DoDochem. Deionized water was used to prepare all aqueous electrolytes.

**Electrolyte preparation:** ZnSO_4_·7H_2_O was dissolved in purified water to create a 2M ZnSO_4_ solution. Different amounts (2 mM, 4 mM, 8 mM,16 mM and 32 mM, respectively) of AChI, ChI and ETMAI were added into 2M ZnSO_4_ electrolyte.

**Preparation of iodine (I_2_) cathodes:** Typically, 0.25 g of iodine powder was mixed with 0.5 g of porous carbon (Kochen black) through grinding for 5 minutes. The resulting mixture was sealed in a glass bottle and heated at 95°C for 6 hours to synthesize the carbon@I_2_ composite. To prepare the cathode slurry for the ZIB full cell, carbon@I_2_ (70 wt%), Super P (20 wt%), and PVDF binder (10 wt%) were dispersed in 1-methyl-2-pyrrolidone (NMP) and stirred for 1 hour to achieve a homogeneous mixture. The slurry was then uniformly coated onto carbon cloth and dried in an oven at 50°C for 6 hours. The content of the active substance iodine of the cathode material was identified by the thermogravimetric analysis (TGA) and the areal iodine loading was controlled at approximately ~1.5 mg cm^-2^.

**Materials Characterization:** Fourier transform infrared were tested by FT-IR (Bruker ALPHA Ⅱ). ^1^H nuclear magnetic resonance spectroscopy (^1^H NMR) was carried out by using AVANCE III 400 MHz equipment. X-ray photoelectron spectroscope (XPS) patterns were obtained by Thermo Scientific K-Alpha (Al Kα radiation). X-ray diffraction (XRD) patterns of the powders and membranes were obtained by a Rigaku SmartLab 9 kW X-ray diffractometer (Cu Kα radiation). Scanning Electron Microscopy (SEM) images were obtained using a scanning electron microscope (Gemini SEM300) operating at 10 kV using tungsten filament as the electron source. The UV-vis spectra were obtained using ultraviolet-visible spectrophotometer (UV-vis, Lambda950). Atomic force microscopy (AFM) tests were conducted on scanning probe microscope (Bruker Dimension ICON). The pH of the electrolytes was measured using the pH meter (PHS-3E). The ionic conductivity was measured via conductivity meter (DOS-307A). Viscosity/Density measurements were tested using Anton Paar (SVM 1101).

**Electrochemical Measurement:** Linear sweep voltammetry (LSV) was carried out at 1 mV s^-1^ in a three-electrode configuration using 1M Na_2_SO_4_ with or without additives, where two separate Pt foils were used as the working and counter electrodes, and Ag/AgCl as the reference electrode. Cyclic voltammetry (CV) measurement was performed in a three-electrode system, employing Ti foil as the working electrode, Zn foil as the counter electrode, and Ag/AgCl as the reference electrode. Linear polarization (Tafel) experiment was conducted in Zn//Zn symmetric cells over a potential range of -0.2 V to 0.2 V (vs. Zn/Zn^2+^) at a scan rate of 10 mV s^-1^. Chronoamperometry (CA) was carried out at a constant overpotential of -200 mV for 150 s, also using Zn//Zn symmetric cells. Electrochemical impedance spectroscopy (EIS) was measured at a fixed amplitude of 5 mV across a frequency range of 0.1 Hz to 1 MHz. The alternating current (A.C.) voltammetry measurement was performed with an amplitude of 5 mV at a fixed frequency of 1000 Hz, covering a potential range from 1.0 to 0.1 V (versus Zn/Zn^2+^). All above measurements were conducted using an electrochemical analyzer (CHI 760D). The electrochemical tests for Zn//Zn symmetric, Zn//Cu asymmetric, and Zn//I_2_ full cells were evaluated using CR2032 coin cells, the glass fiber filters (GF/F, Whatman) were used as separators. For symmetric cells, bare Zn foils were employed as both electrodes. In asymmetric cells, Cu foils served as cathodes, while in full cells, I_2_ cathodes replaced Cu foils. The performance metrics of all cell configurations were evaluated using a multichannel battery test system (LAND CT3002A).

**Computational detail:** Classical molecular dynamics (MD) simulations were performed for various electrolytes using the GROMACS 2020.6^[1]^ software, adopting parameters from the General Amber force fields (GAFF). The MD parameters for SO_4_^2-^, ACh^+^, Ch^+^, and ETMA^+^ were generated using ACPYPE^[2]^, and the corresponding atom charges were based on RESP charges^[3]^ fitting via the antechamber tool^[4]^. To approximate the effect of charge transfer and polarizability in the bulk phase, the partial charges of SO_4_^2-^, ACh^+^, Ch^+^, and ETMA^+^ were scaled by a constant of 0.75.^[5]^ The H_2_O molecules were described using the simple point charge extended (SPC/E) model. The numbers of salt and water molecules used in the simulations are listed in Table S1. The energy minimization was conducted using the steepest descent algorithm with a force tolerance of 500 kJ mol^-1^ nm^-1^. Periodic boundary conditions were imposed in all three directions. Following energy minimization, an NPT ensemble simulation was carried out for 20 ns at 298.15 K to equilibrate the system. The pressure was maintained isotropically at 1 bar using the Berendsen barostat^[6]^ and the temperature was controlled at 298.15 K using the V-rescale thermostat^[7]^. The Particle Mesh Ewald (PME) method^[8]^ was used for long-range electrostatic interactions, while the Lennard-Jones potential described intermolecular van der Waals interactions. A cutoff length of 1.2 nm was applied for both electrostatic and vdW interactions. The integration time step was 2 fs. A subsequent 20 ns NVT ensemble simulation was performed for data analysis. The radial distribution functions (RDFs) were calculated using the in-built module, and the snapshots from MD simulation were rendered with VMD software.^[9]^

The ESP mapping, the binding energies/adsorption energies and LUMO-HOMO energy level were calculated by the density functional theory (DFT) calculations. The structure optimization was performed at M0-62X/def2-TZVP level and DFT-D3 dispersion correction. The exchange-correlation interactions were described using the Perdew-Burke-Ernzerhof (PBE) functional within the generalized gradient approximation (GGA). The plane wave basis was set with an energy cutoff of 500 eV for all structural optimizations and self-consistent calculations. The vacuum region was set to 15 Å. During structural optimization, the bottom two layers of atoms were kept fixed, while the top two layers were allowed to relax. The structures were relaxed until the convergence criteria for the iteration in self-consistent field (SCF) is set to be 10^-5^ eV, and the force convergence criterion for atomic relaxation is set to be 0.02 eV Å^-1^. Van der Waals interactions were accounted for using the DFT-D3 method. The adsorption energies (E_ad_) of Zn atom, H_2_O molecule, ACh^+^, Ch^+^ and ETMA^+^ on different planes of Zn were calculated with the following equation.

$E_{ad}=E_{total}-E_{x}-E_{sub}$ (1)

where *E_total_*, *E_x_*, and E*_sub_* are the energies of the whole system, Zn atom, H_2_O molecule, ACh^+^, Ch^+^or ETMA^+^ and the Zn metal substrate, respectively.


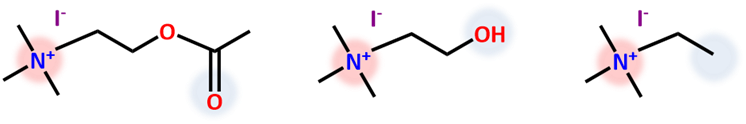


**AChI ChI ETMAI**

Figure S1. Molecular structures of different additives.


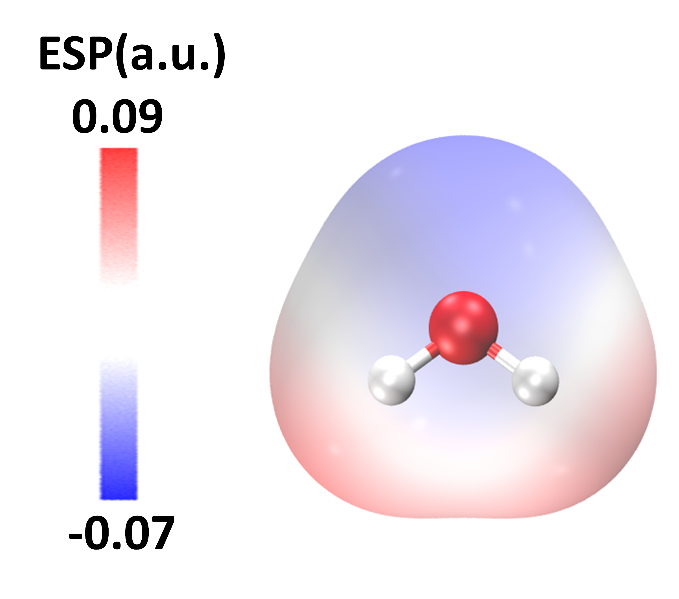


Figure S2. ESP mapping of H_2_O.


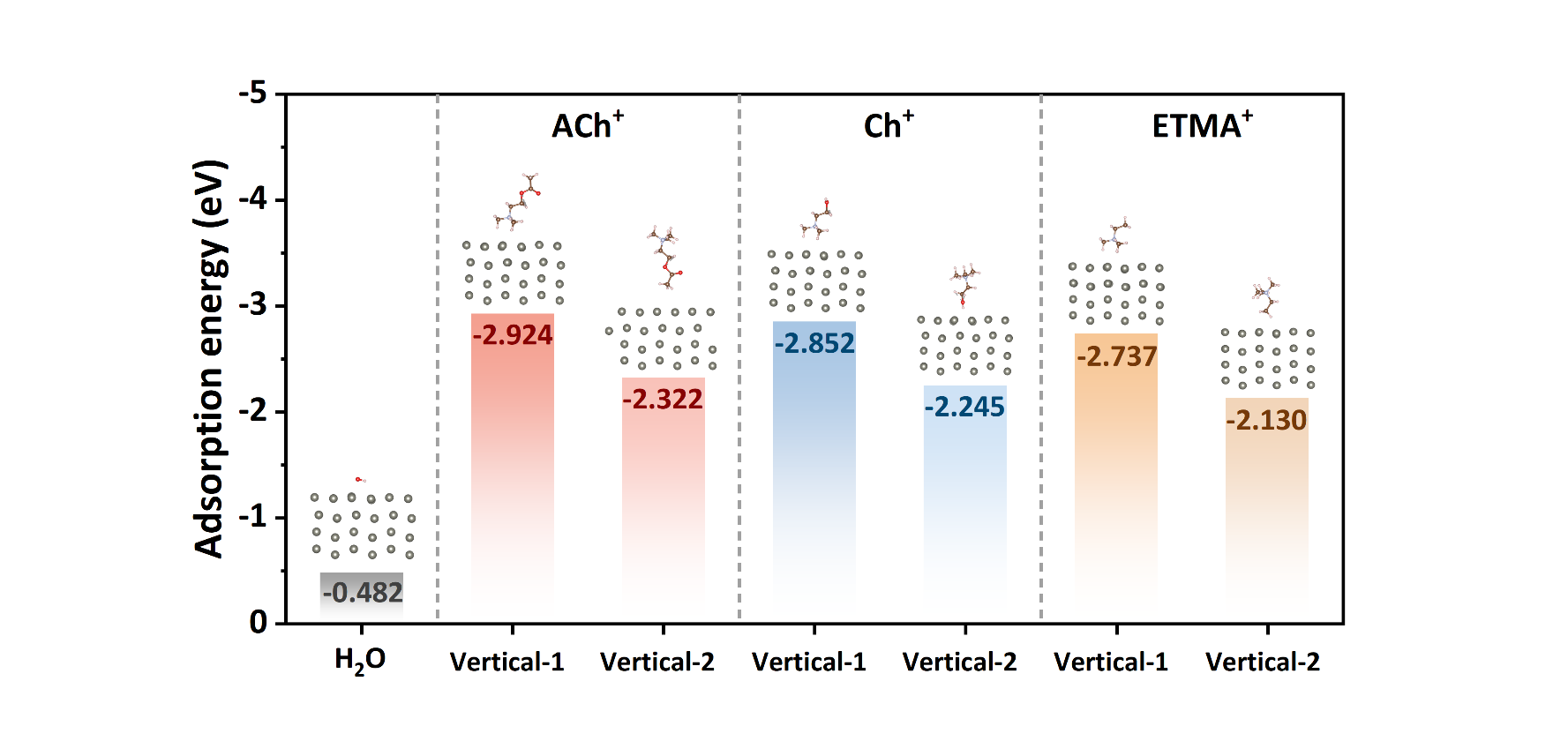


Figure S3. Calculated adsorption energies of H_2_O and additives in two vertical states on the Zn (100) plane.


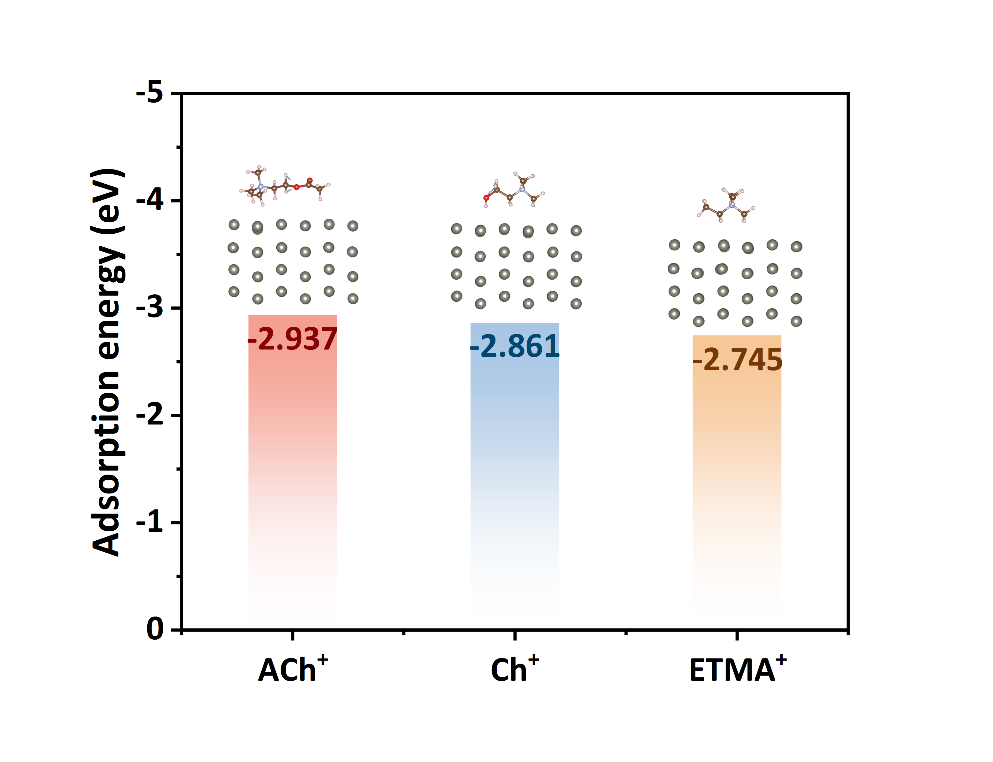


Figure S4. Calculated adsorption energies of additives in horizontal state on the Zn (100) plane.


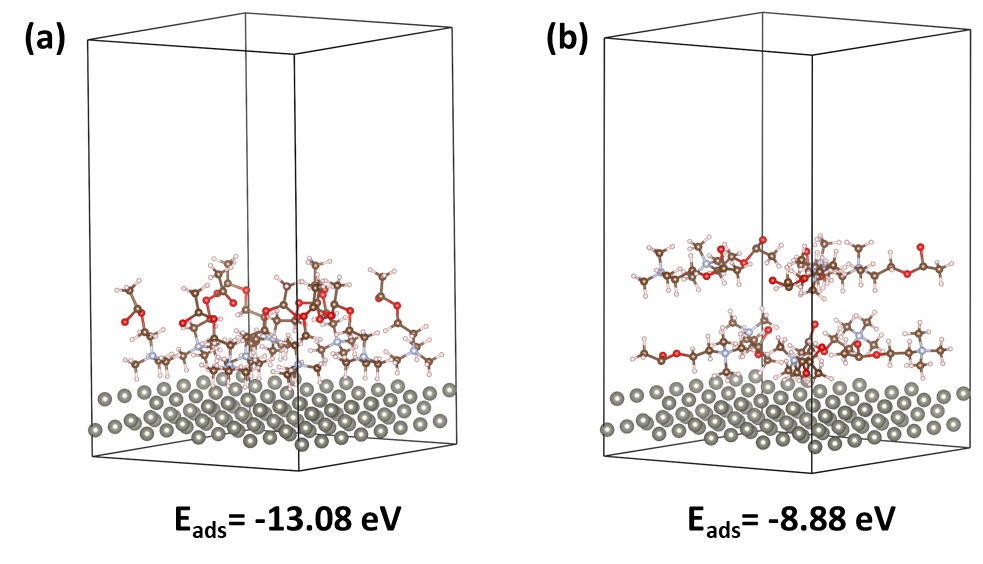


Figure S5. Calculation models of ACh^+^(a)verticle and (b) parallel absorbed on Zn (100) plane.


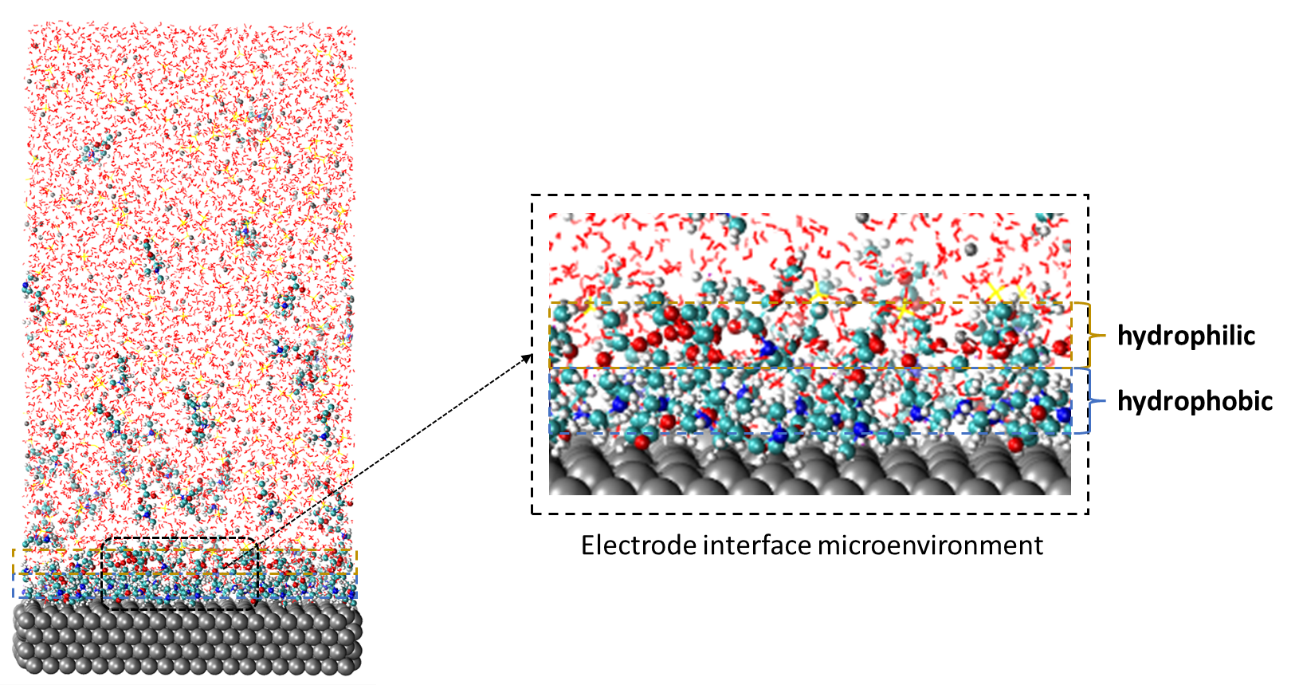


Figure S6. MD simulation snapshots of AChI-ZnSO_4_ electrolyte with Zn anode.


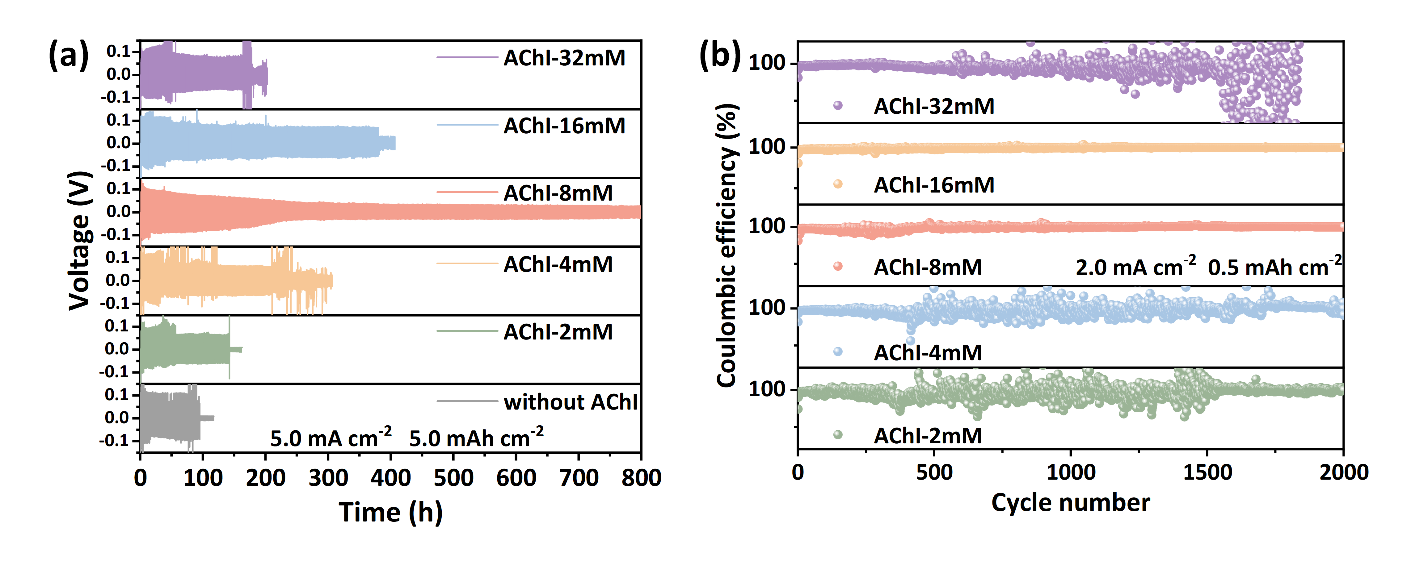


Figure S7. (a) Long-term cycling performances of Zn//Zn symmetrical cells at 5.0 mA cm^-2^ and 5.0 mAh cm^-2^, and (b) CE performances of Zn//Cu asymmetric cells at 2.0 mA cm^-2^ and 0.5 mAh cm^-2^ using 2M ZnSO_4_ and AChI-ZnSO_4_ electrolytes (2, 4, 8, 16, and 32 mM, respectively).

<Note>

By comparing the cycle life in Zn//Zn symmetric cells and the coulombic efficiency (CE) in Zn//Cu asymmetric cells, the optimal AChI concentration is determined to be 8 mM (Figure S7).


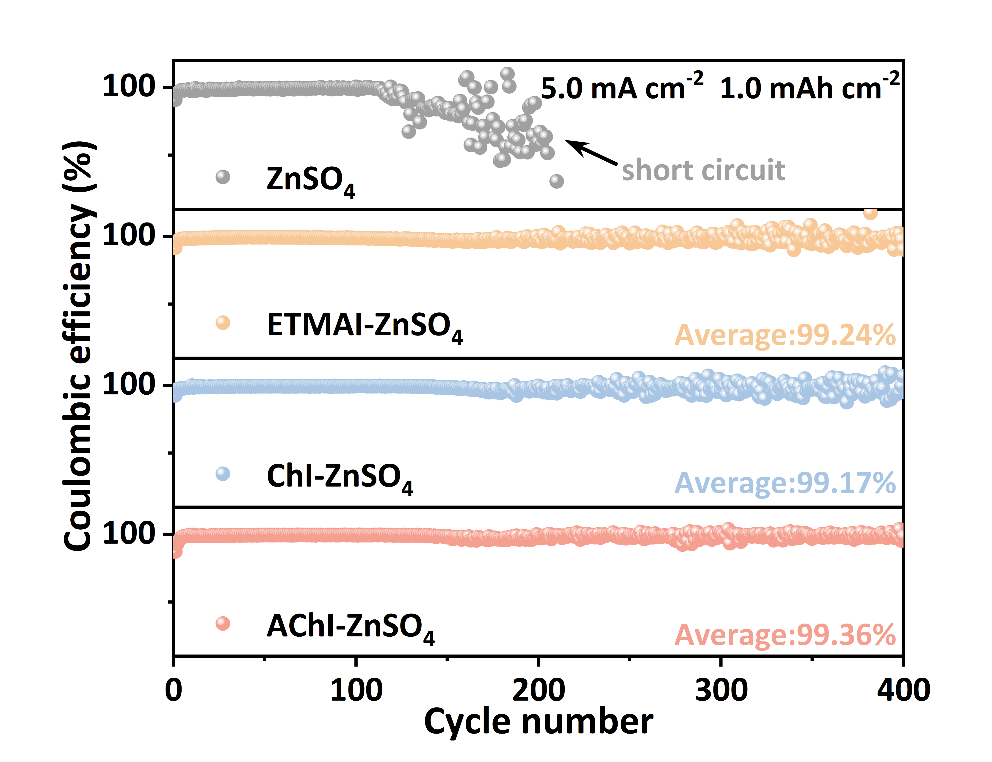


Figure S8. CE performances of Zn//Cu asymmetric cells with different electrolytes at 5.0 mA cm^-2^ and 1.0 mAh cm^-2^.


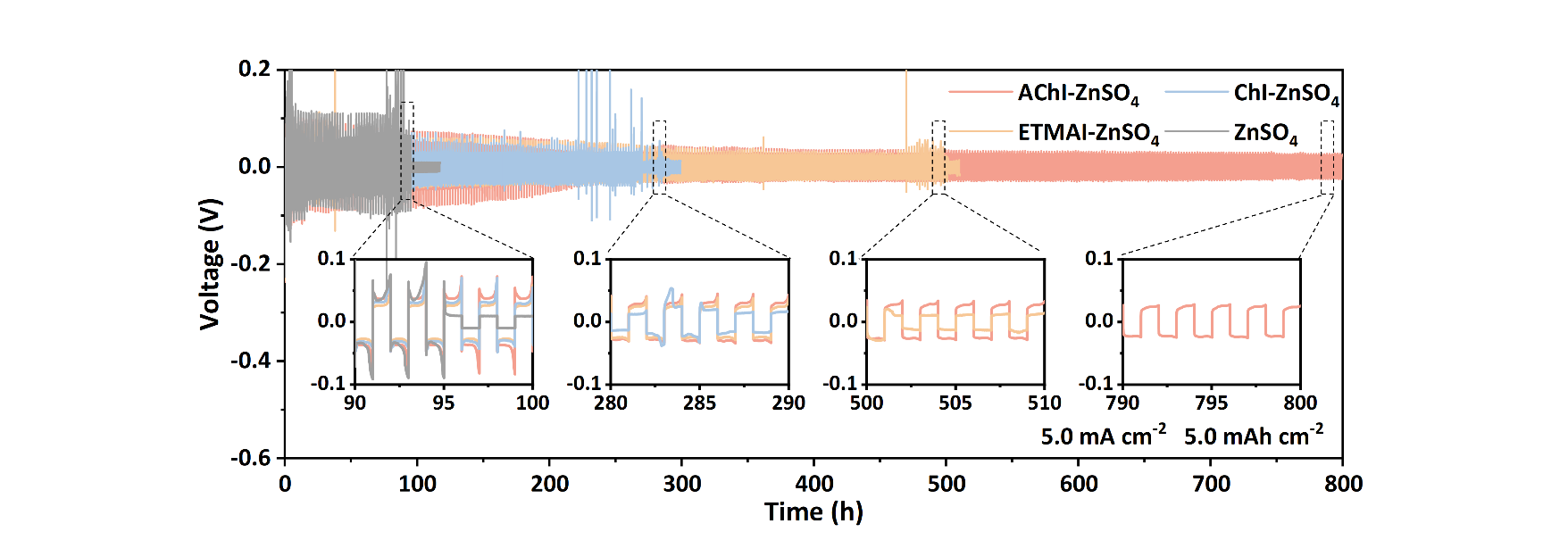


Figure S9. Long-term cycling performances of Zn//Zn symmetrical cells using different electrolytes at 5.0 mA cm^-2^ and 5.0 mAh cm^-2^.

<Aanalysis for Figure S8 and Figure S9>

As shown in Figure S8, Zn//Cu cells with hybrid electrolytes demonstrate stable cycling for 400 cycles at a high current density of 5.0 mA cm^-2^, with average CE of 99.36%, 99.17%, and 99.24% for AChI-ZnSO_4_, ChI-ZnSO_4_, and ETMAI-ZnSO_4_, respectively. While cells with ZnSO_4_ electrolyte fail after 180 cycles, exhibiting significant fluctuations throughout the cycling process. Furthermore, the Zn//Zn symmetric cells with AChI-ZnSO_4_ exhibit a cycle life of over 800 h at 5.0 mA cm^-2^ and 5.0 mAh cm^-2^, while ChI-ZnSO_4_ and ETMAI-ZnSO_4_ achieve cycle lives of 280 h and 500 h under the same conditions, respectively (Figure S9). In comparison, ZnSO_4_ cells short-circuit after only 90 h of cycling.


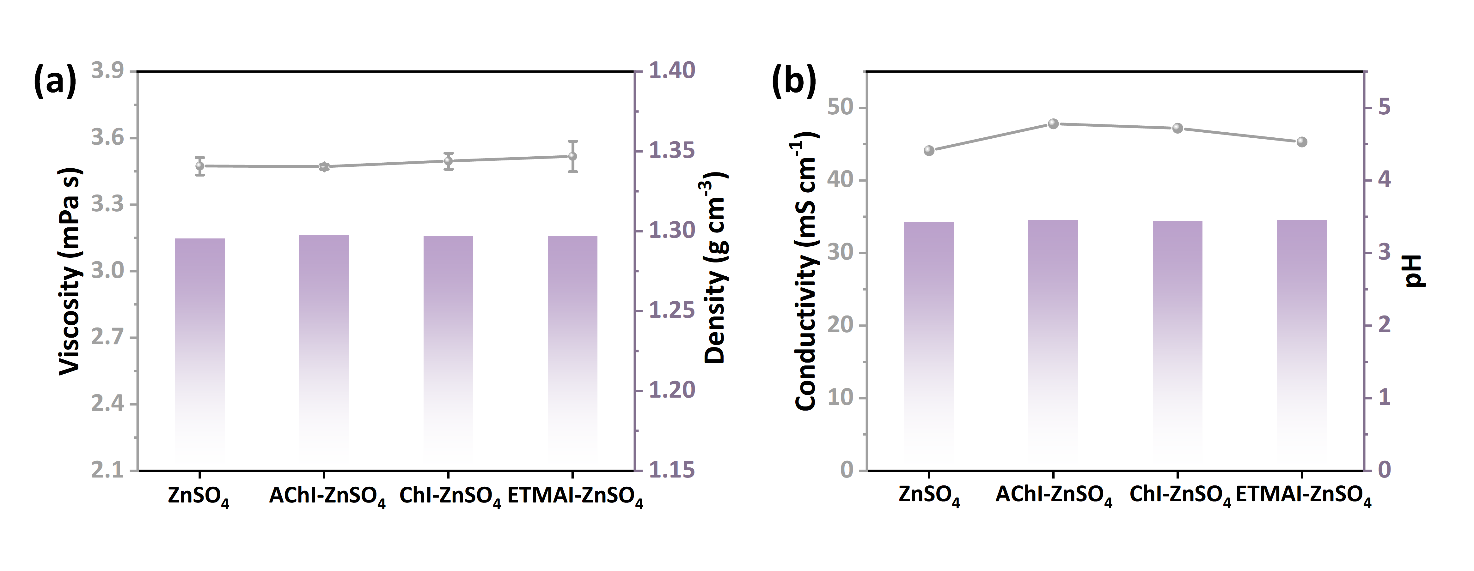


Figure S10. (a)Viscosity and density, (b) ionic conductivities and pH of different electrolytes.


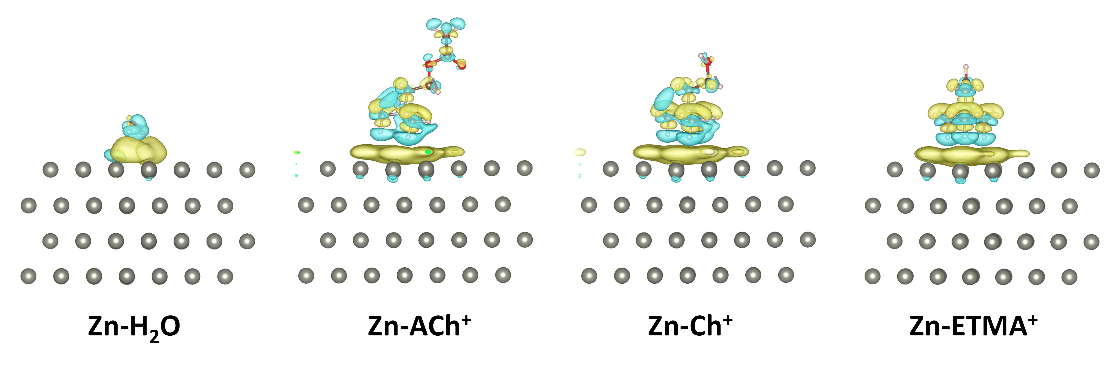


Figure S11. The charge density difference value of H_2_O, ACh^+^, Ch^+^, and d) ETMA^+^ (yellow and cyanine semitransparent clusters represent increase and decrease of electron density, respectively).

Figure S12. The XPS spectra for N1s of Zn surface that is post-soaked and subsequent cleaned with deionized water in different electrolytes.


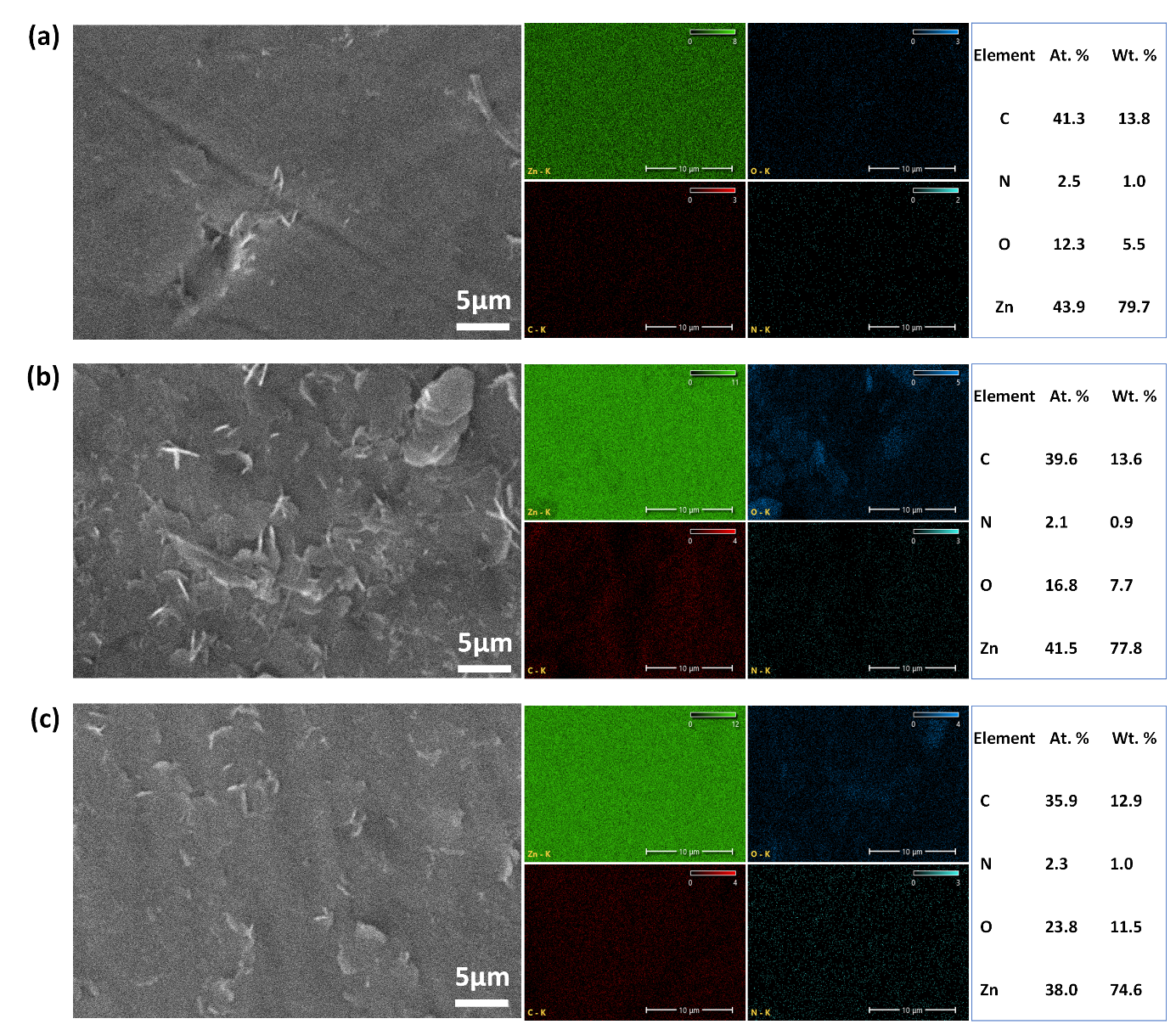


Figure S13. SEM image and EDS mapping of the Zn foil after soaking in different electrolytes, (a) AChI-ZnSO_4_, (b) ChI-ZnSO_4_, and (c)ETMAI-ZnSO_4_.


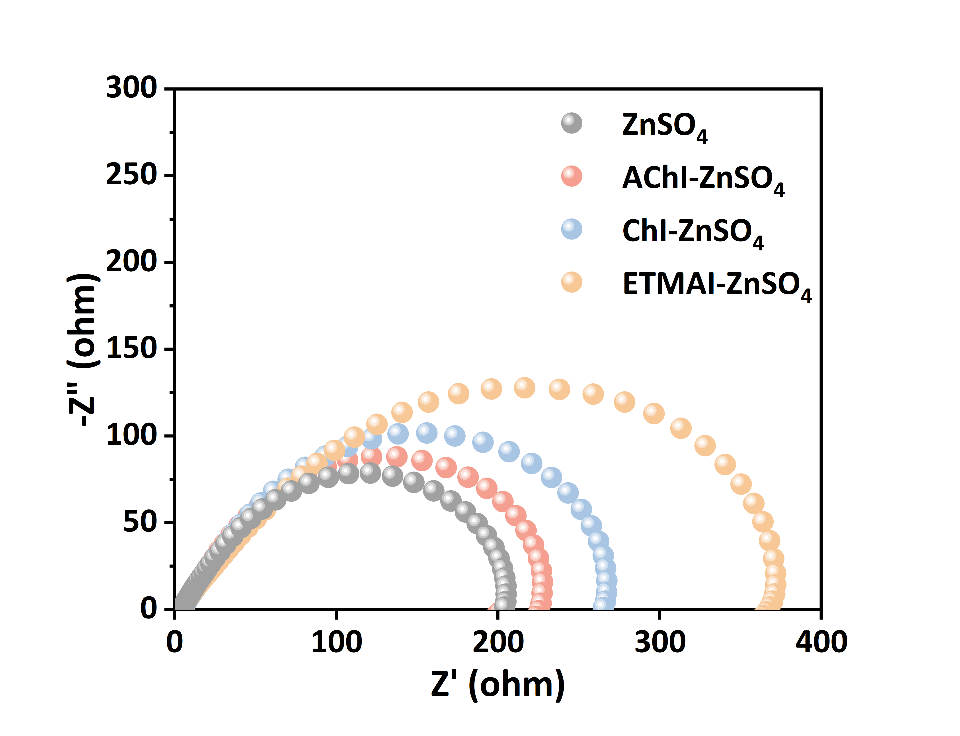


**Figure S14.** Nyquist plots of Zn//Zn symmetric cells with different electrolytes.


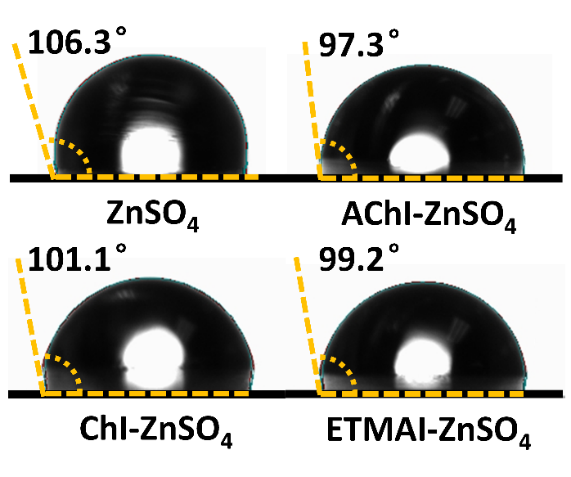


Figure S15. Contact angle measurements of different electrolytes with Zn foil.


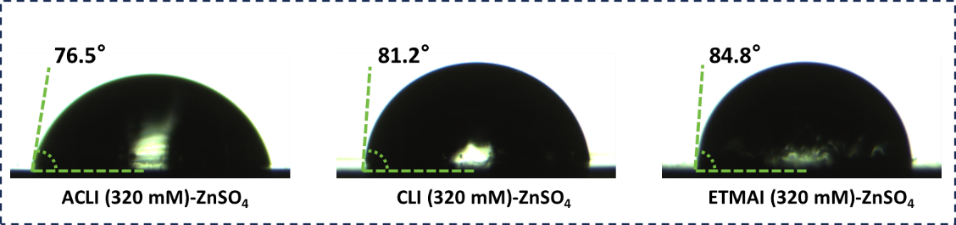


Figure S16. Contact angle measurements of Zn foil in electrolytes with elevated additive concentration (320 mM).

<Note>

To further illustrate the additive’s interfacial affinity, we increased its concentration to 320 mM.


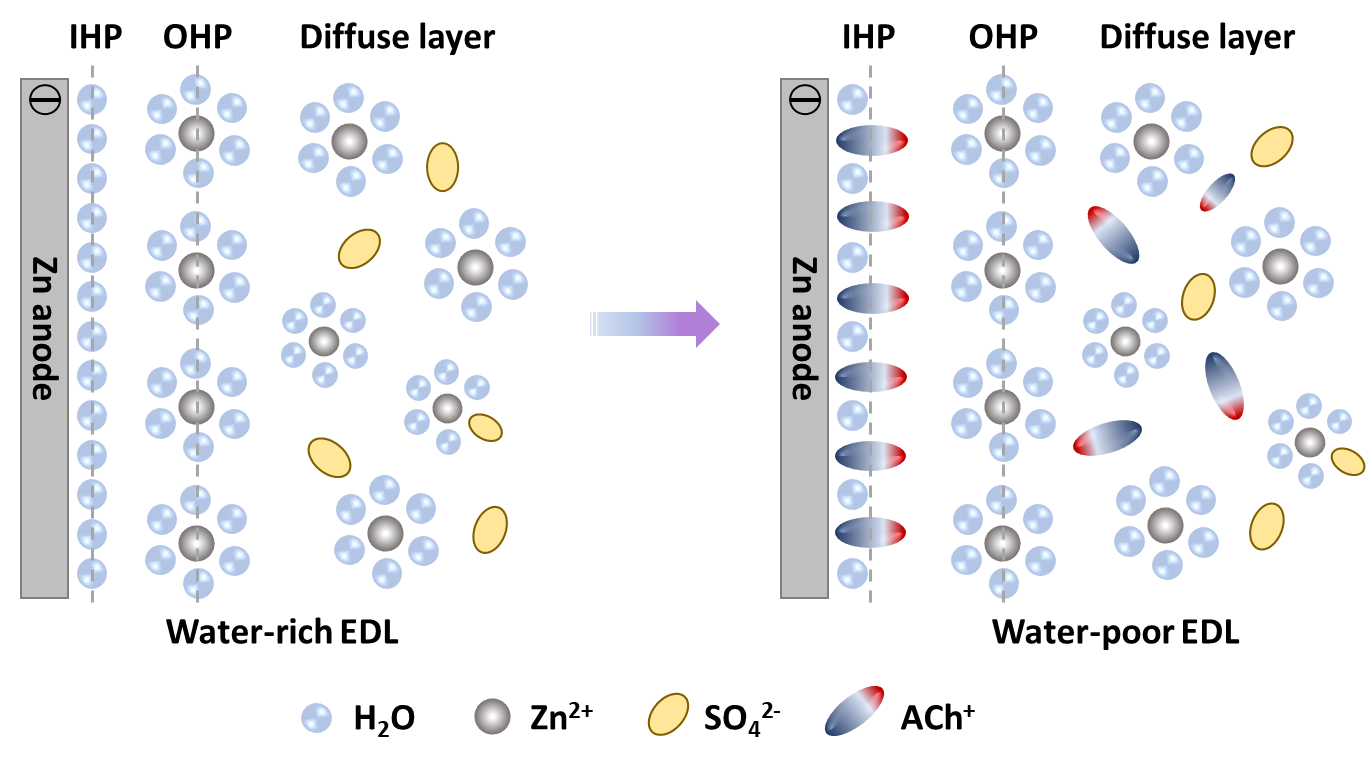


Figure S17. Schematic illustrations of EDL structure in ZnSO_4_ (left) and AChI-ZnSO_4_ (right).


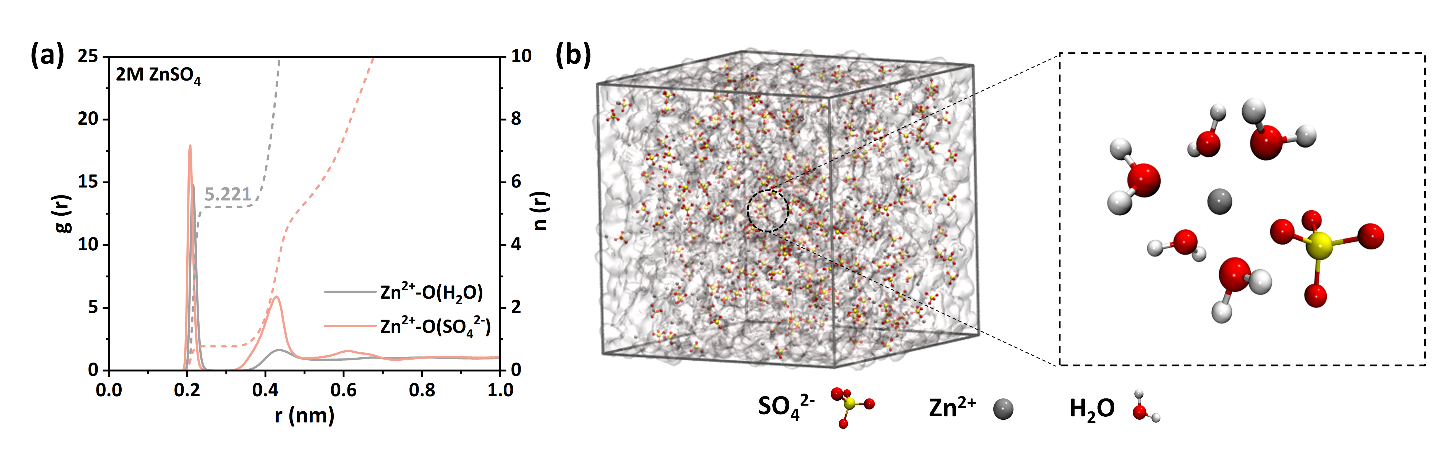


Figure S18. (a) Radial distribution function g(r) and coordination number n(r) for ZnSO_4_ electrolyte. (b) 3D snapshot of ZnSO_4_ and partial enlarged images representing Zn^2+^ solvation structure obtained from MD simulations.


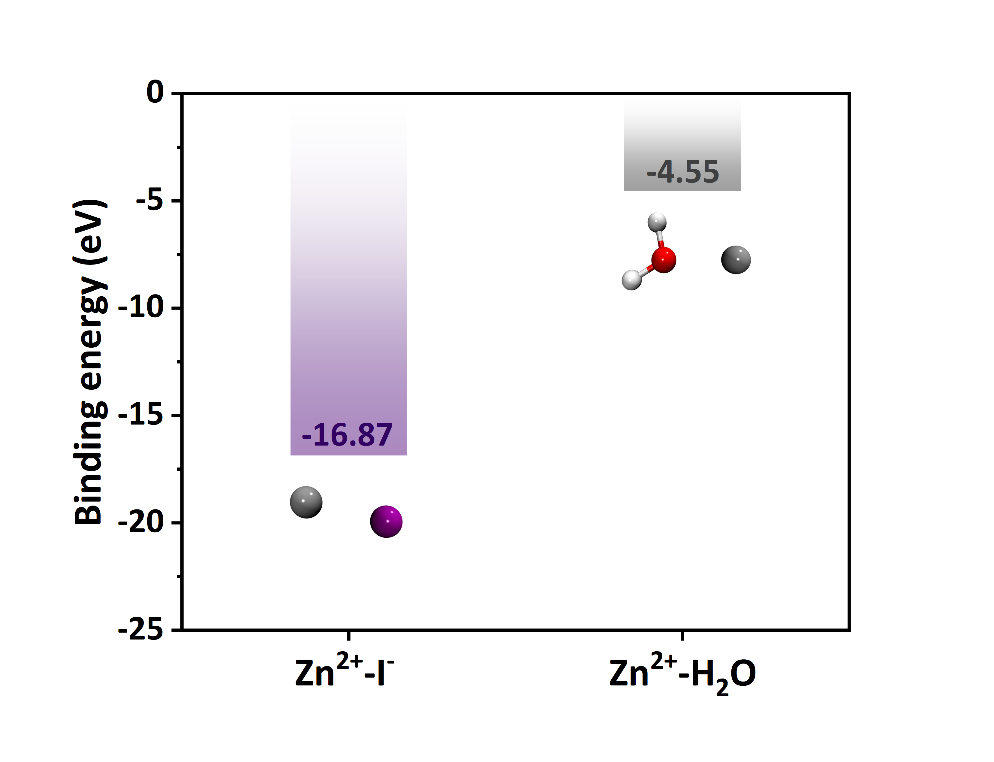


Figure S19. Binding energy of Zn^2+^-I^-^ and Zn^2+^-H_2_O.


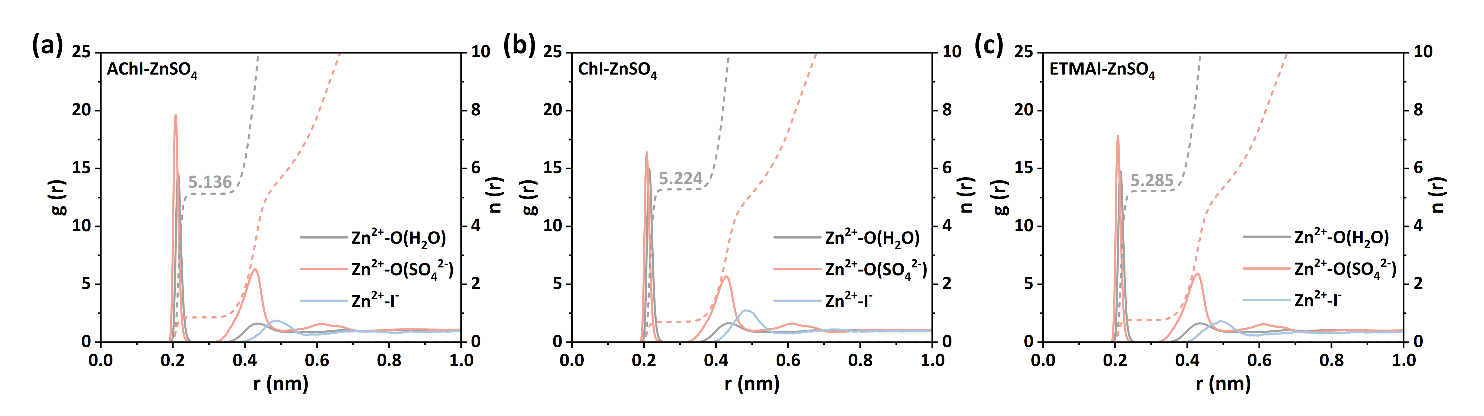


Figure S20. Radial distribution function g(r) and coordination number n(r) for (a) AChI-ZnSO_4_, (b) ChI-ZnSO_4_, and (c) ETMAI-ZnSO_4_ electrolytes.


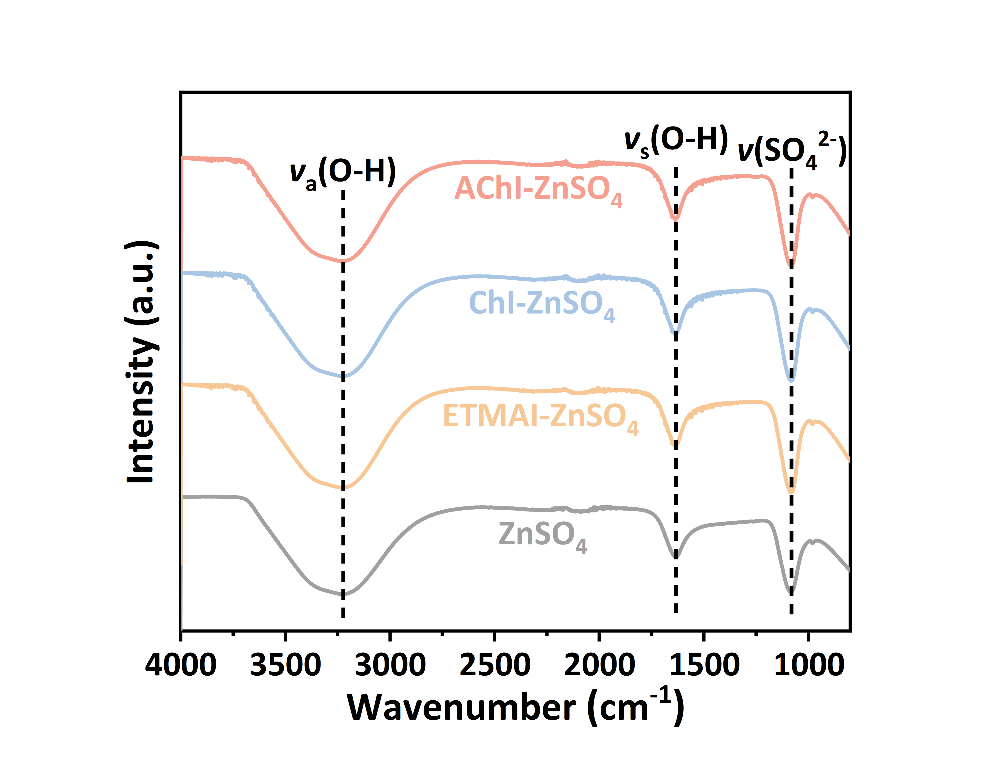


Figure S21. FTIR spectra of different electrolytes.


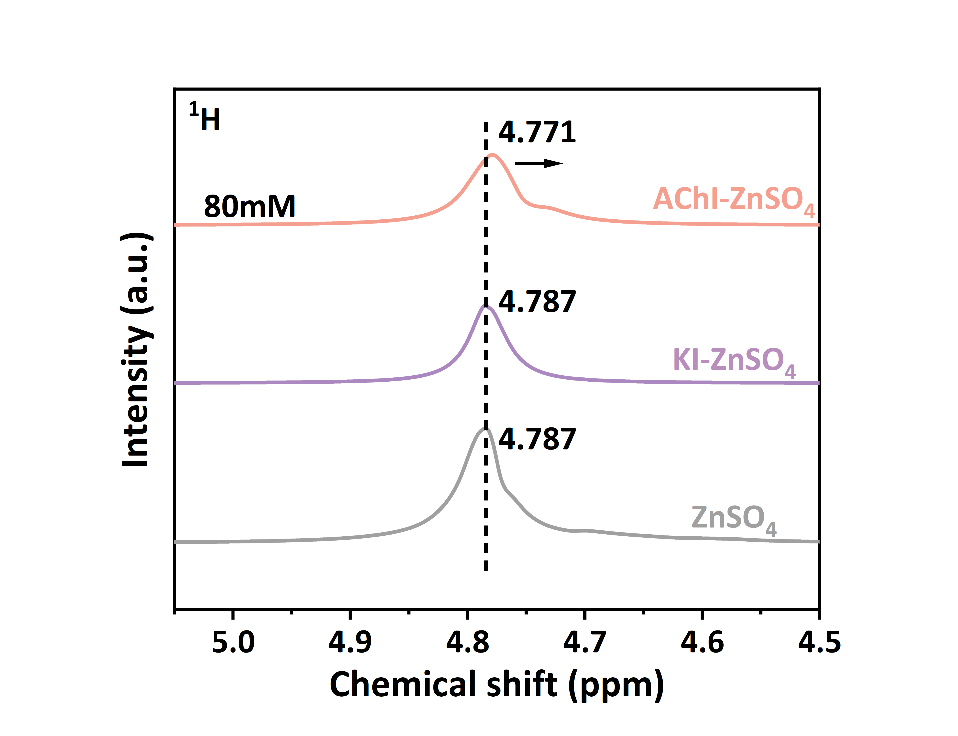


Figure S22. ^1^H NMR spectra of ZnSO_4_, KI (80 mM)-ZnSO_4_ and AChI (80 mM)-ZnSO_4_ electrolytes.


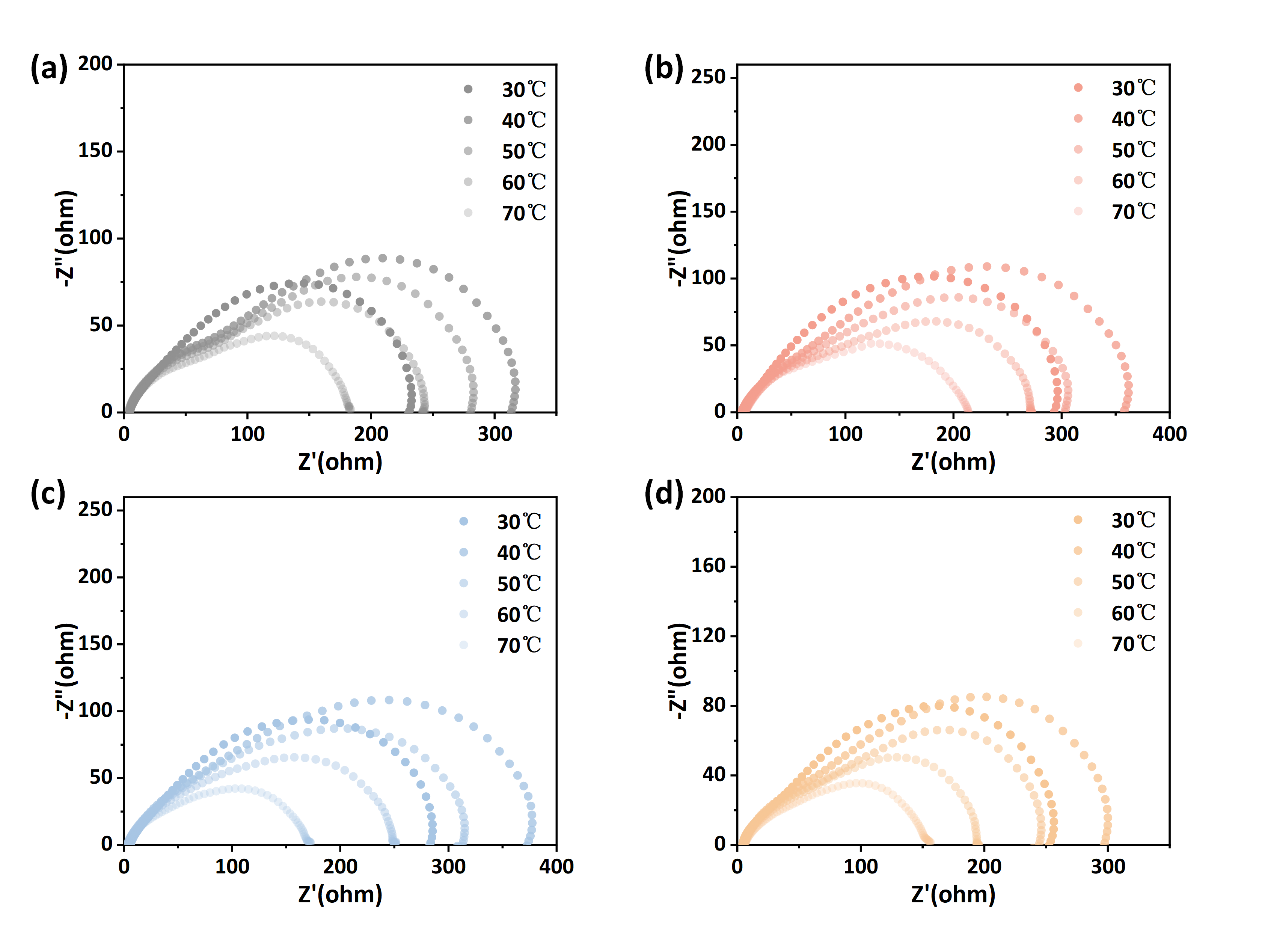


Figure S23. Nyquist plots at different temperatures for (a) ZnSO_4_, (b) AChI-ZnSO_4_, ChI-ZnSO_4_, and (d) ETMAI-ZnSO_4_ electrolytes.

<Note>

The Arrhenius activation energy (*Ea*) can be quantitatively estimated based on the following Arrhenius formula (Figure 2f and Figure S23):

$1/{R_{ct}=A_{exp}(-{E_{a}}/{RT})}$ (2)

where $R_{ct}$ is the interfacial resistance, A is the Arrhenius constant, R is the gas constant, and T is the absolute temperature.^[10]^


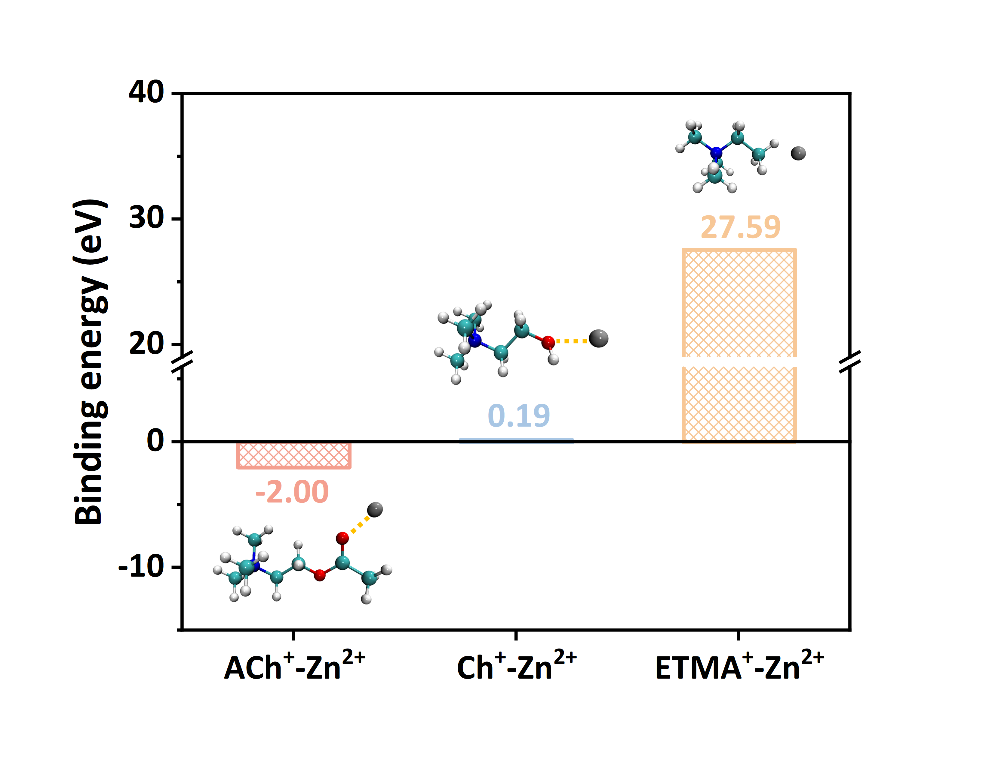


Figure S24. Binding energy of ACh^+^, Ch^+^, and ETMA^+^ with Zn^2+^.


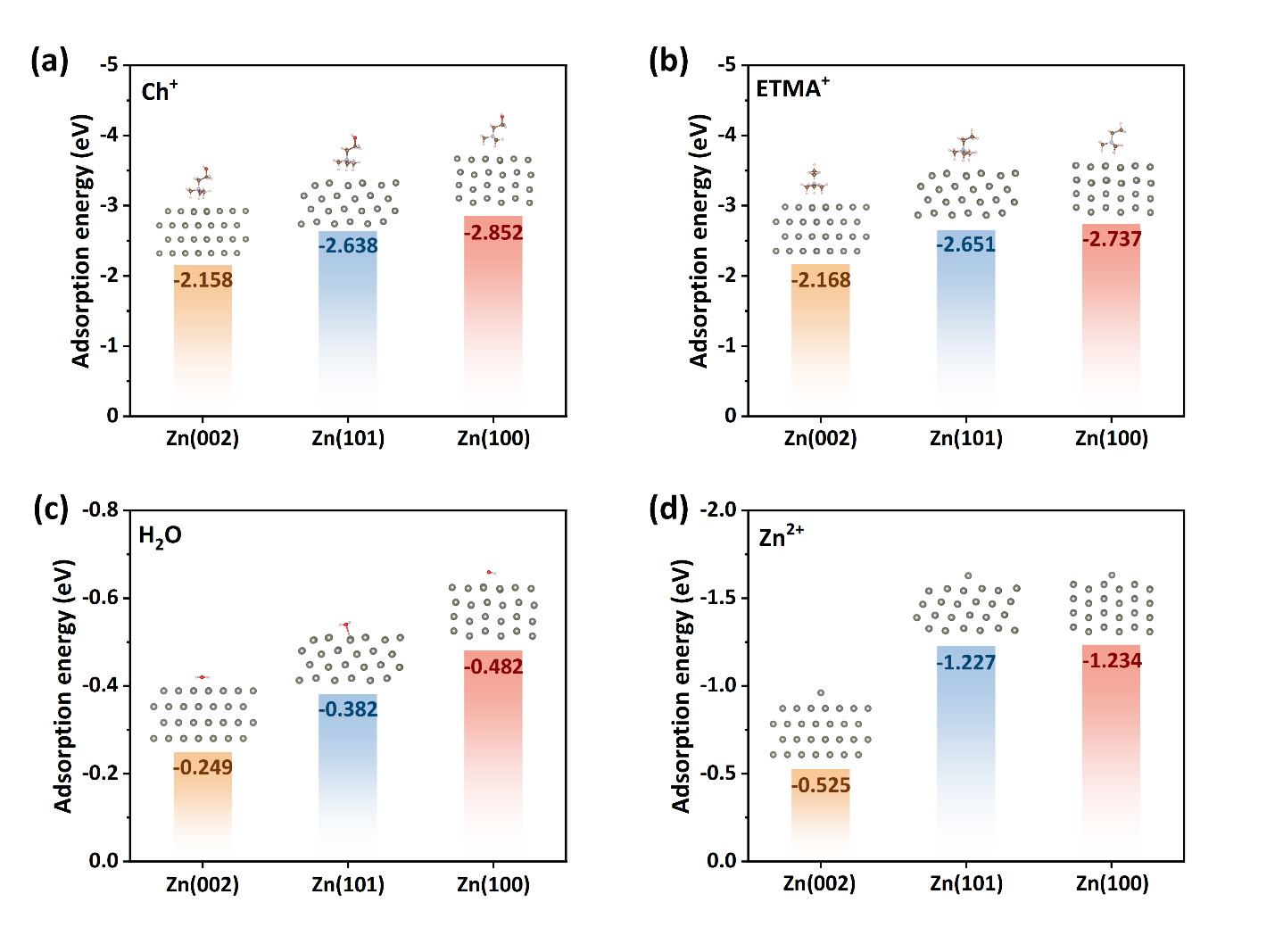


Figure S25. Adsorption energy of (a) Ch^+^, (b) ETMA^+^, (c) H_2_O and (d) Zn^2+^ on different crystal planes of Zn.


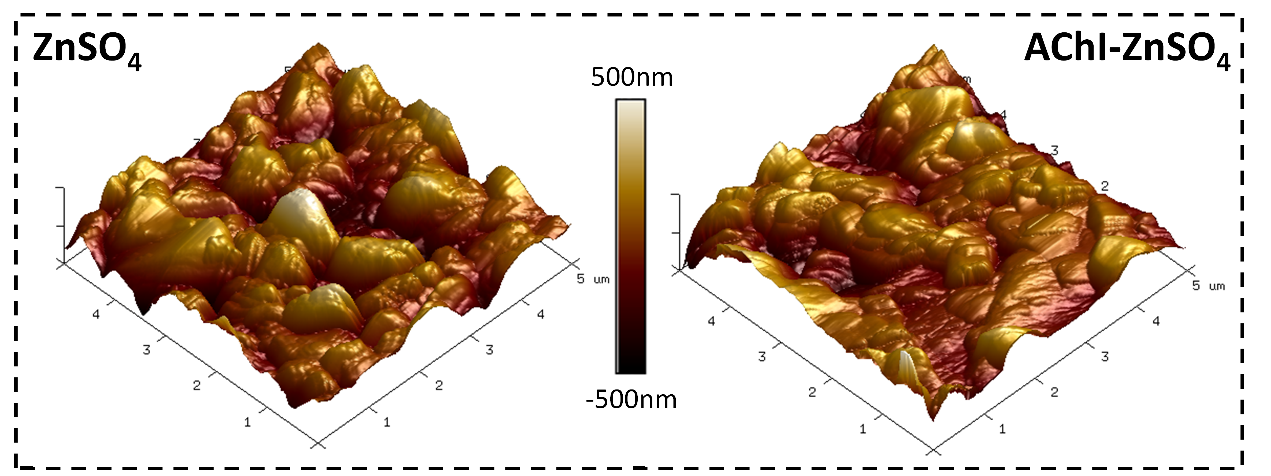


Figure S26. AFM images of Zn anodes in ZnSO_4_ (left) and AChI-ZnSO_4_ (right) electrolytes after 50 cycles at 1.0 mA cm^-2^ and 1.0 mAh cm^-2^.


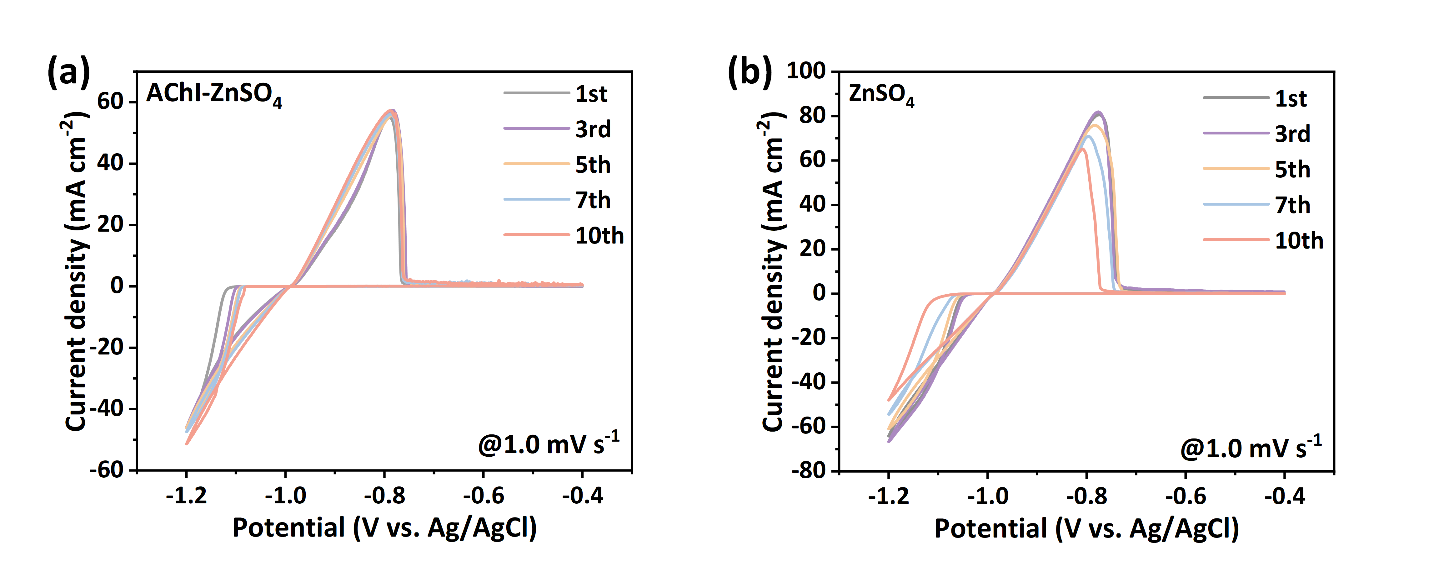


Figure S27. CV profiles of Zn plating/stripping with Ti foil as working, Zn as counter, and Ag/AgCl as reference electrode at 1.0 mV s^−1^ in (a) AChI-ZnSO_4_ and (b) ZnSO_4_ electrolytes.


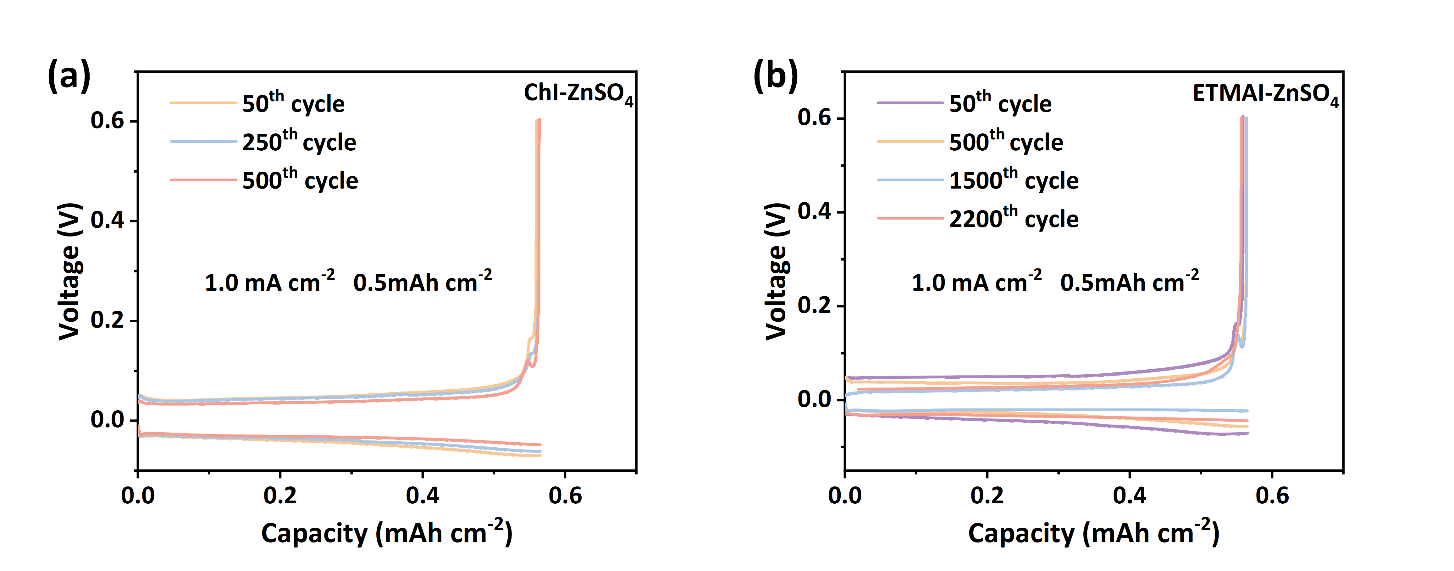


Figure S28. Voltage profiles of Zn//Cu asymmetric cells at various cycles in (a) ChI-ZnSO_4_ and (b) ETMAI-ZnSO_4_ electrolytes.


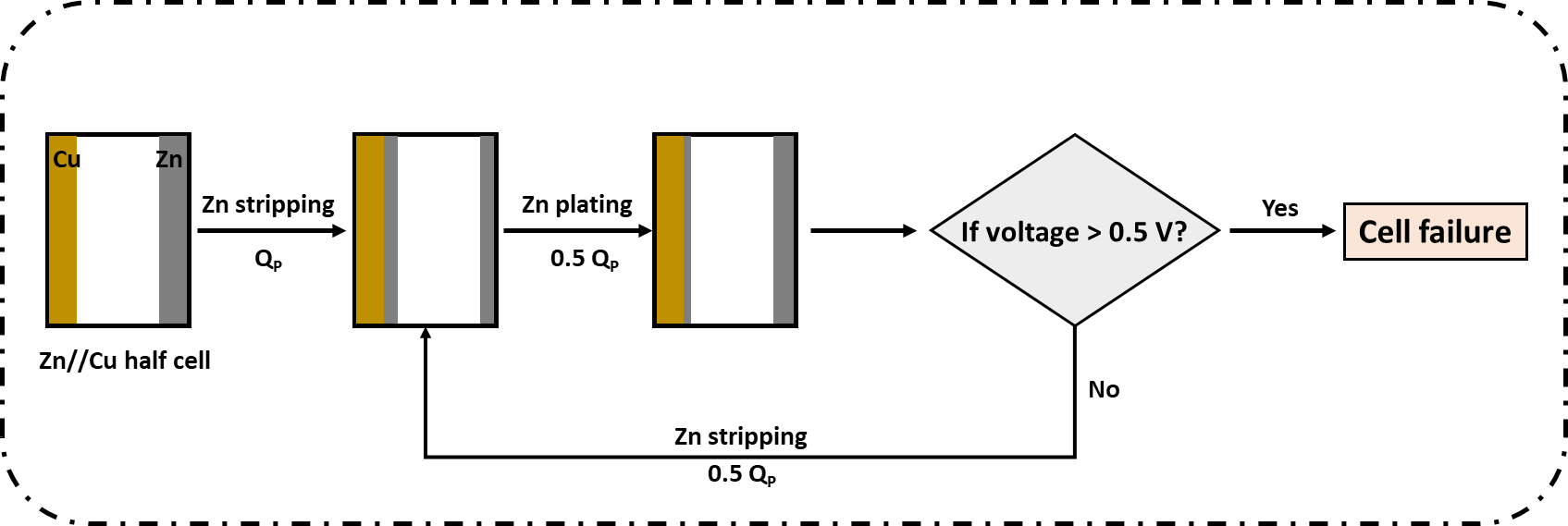


Figure S29. Flowchart of the proposed “pre-activated mode” galvanostatic protocol for evaluating the reversibility of Zn plating/stripping at 50% DOD_Zn_. An area of Zn is plated on a copper substrate as a Zn storage layer (Q_p_). A certain areal capacity of Zn deposited as Zn reservoir (Q_p_) is plated on Cu substrate. The Zn//Cu cell is then subjected to Zn plating/stripping at a fixed areal capacity (0.5 Q_p_) at the current density of 2.0 mA cm^-2^ until the voltage at stripping reaches a preselected upper cut-off voltage of 0.5 V.^[11]^


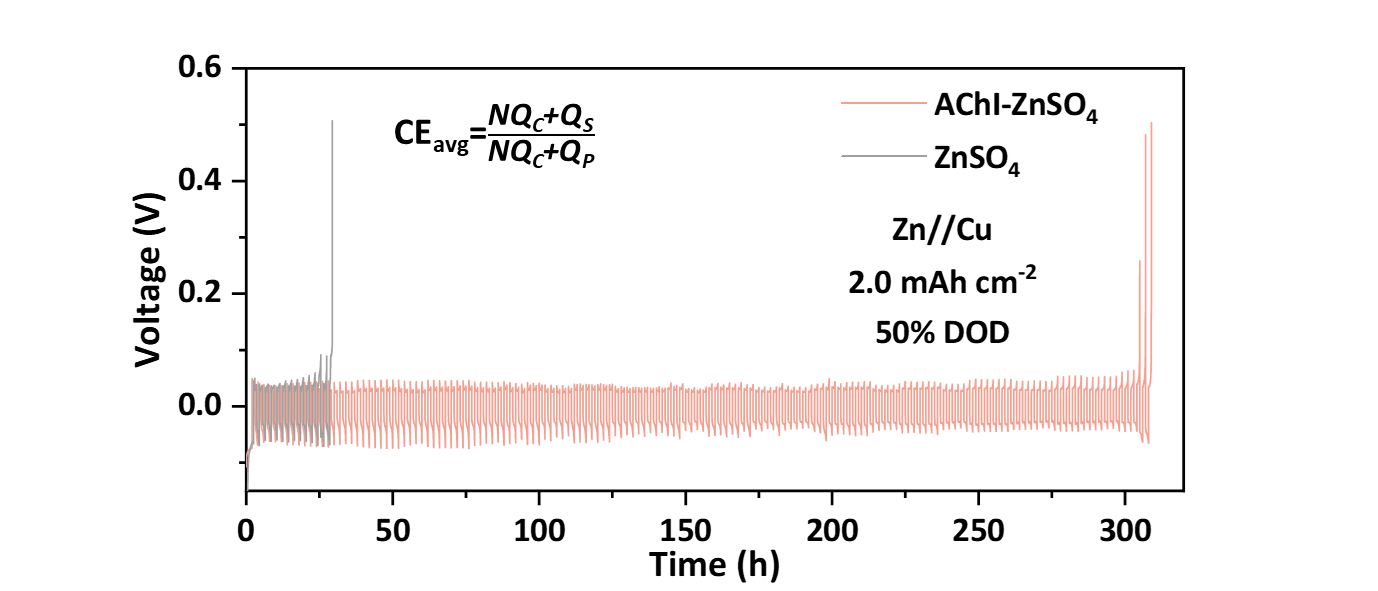


Figure S30. Cycling performances of Zn//Cu half-cells with/without AChI at a current density of 2.0 mA cm^-2^ and 50% DOD_Zn_ with areal capacities of 2.0 mAh cm^-2^.

<Note>

Based on the calculation formula provided in the figure, the average CE of the AChI-containing electrolyte is 99.34%, compared to 94.33% for the bare ZnSO_4_ electrolyte.


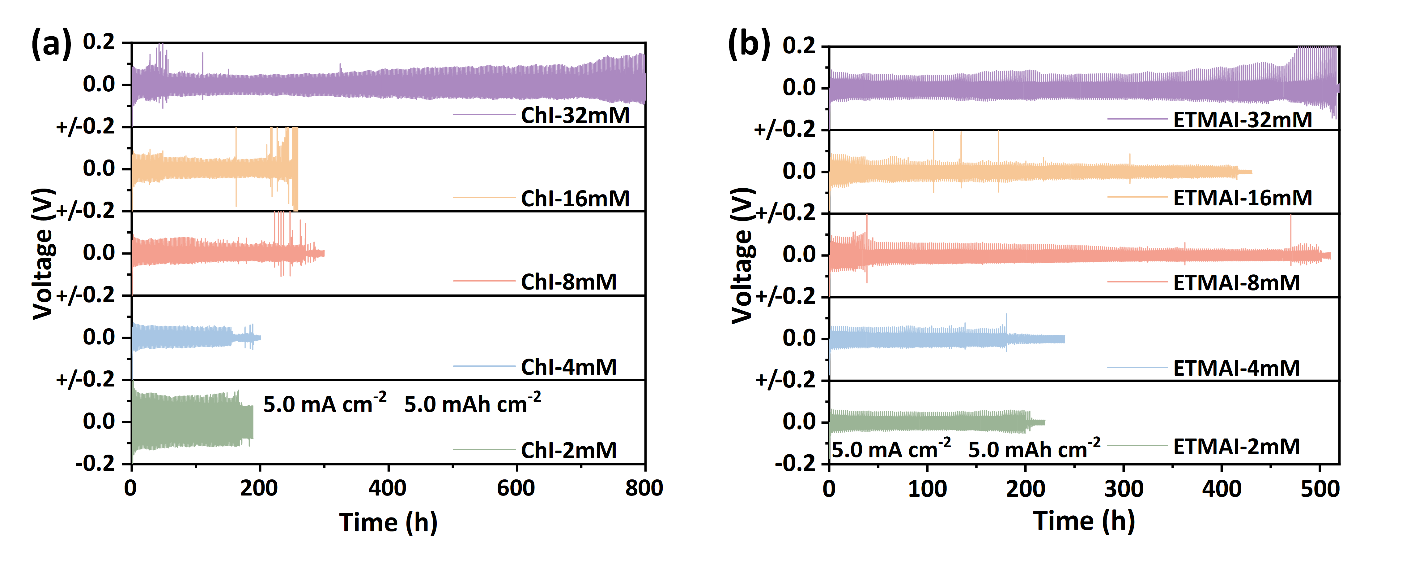


Figure S31. Long-term cycling performances with (a) ChI (2, 4, 8, 16, and 32 mM, respectively) and (b) ETMAI (2, 4, 8, 16, and 32 mM, respectively) in 2M ZnSO_4_ electrolyte of Zn//Zn symmetrical cells at 5.0 mA cm^-2^ and 5.0 mAh cm^-2^.


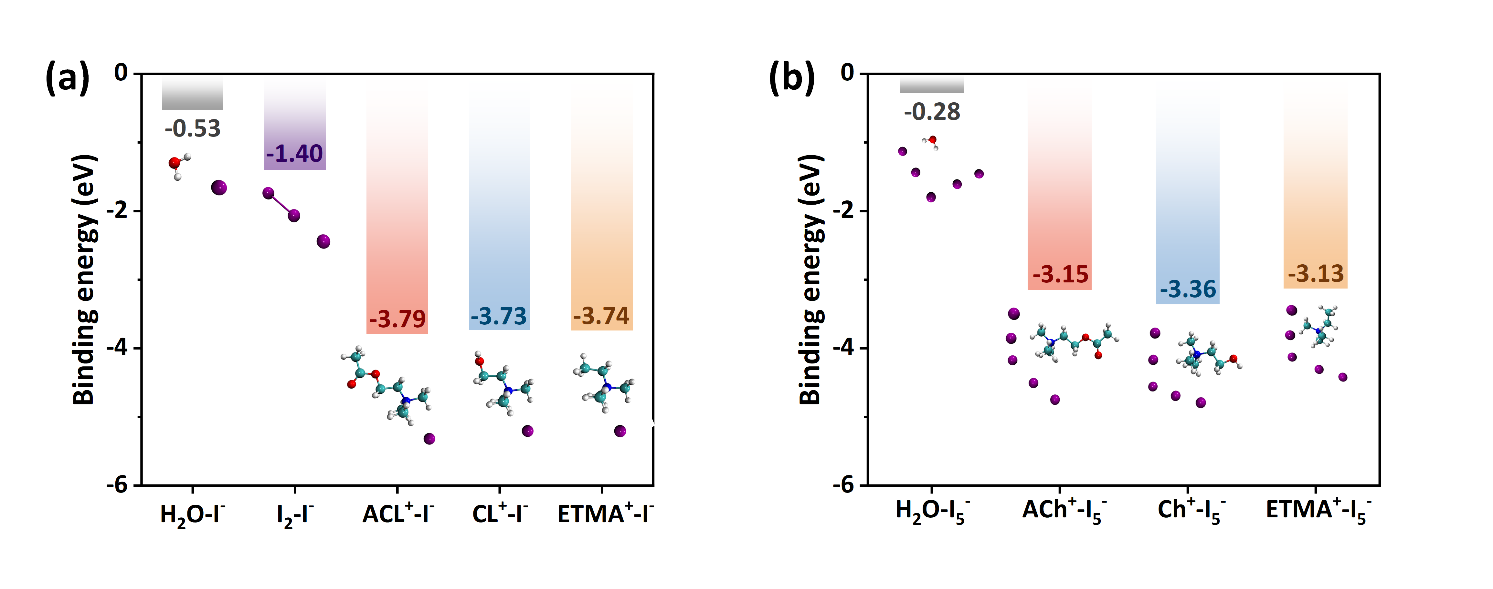


Figure S32. Binding energy of (a) H_2_O-I^-^, I_2_-I^-^, ACh^+^-I^-^, Ch^+^-I^-^ and ETMA^+^-I^-^ and (b) H_2_O-I_5_^-^, ACh^+^-I_5_^-^, Ch^+^-I_5_^-^ and ETMA^+^-I_5_^-^.


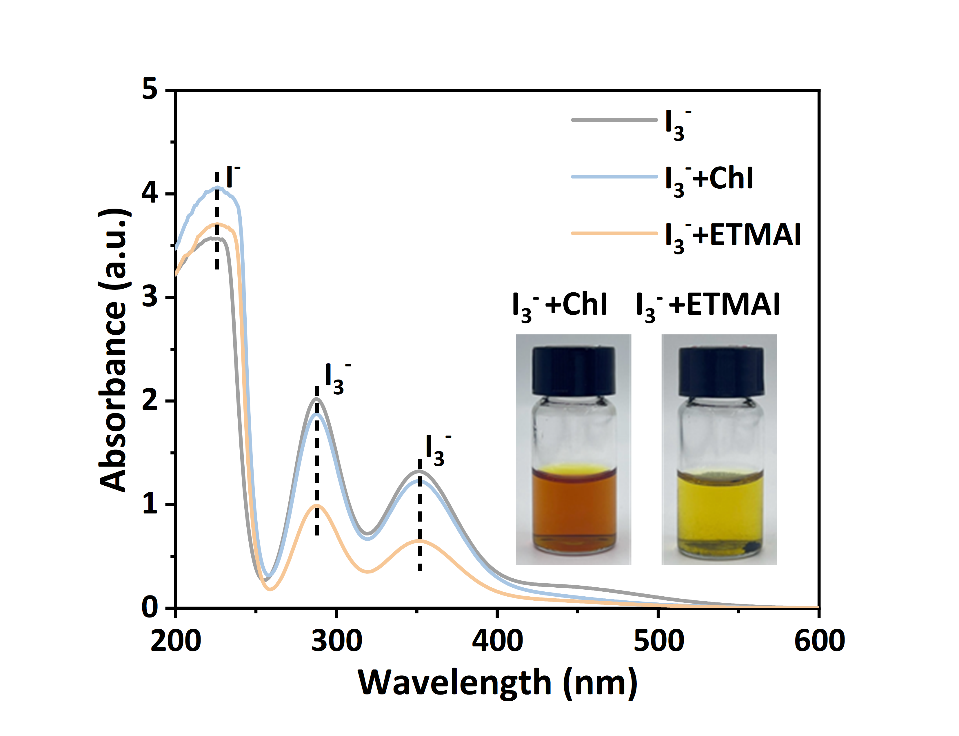


Figure S33. UV-vis spectra of polyiodide solutions with ChI and ETMAI.


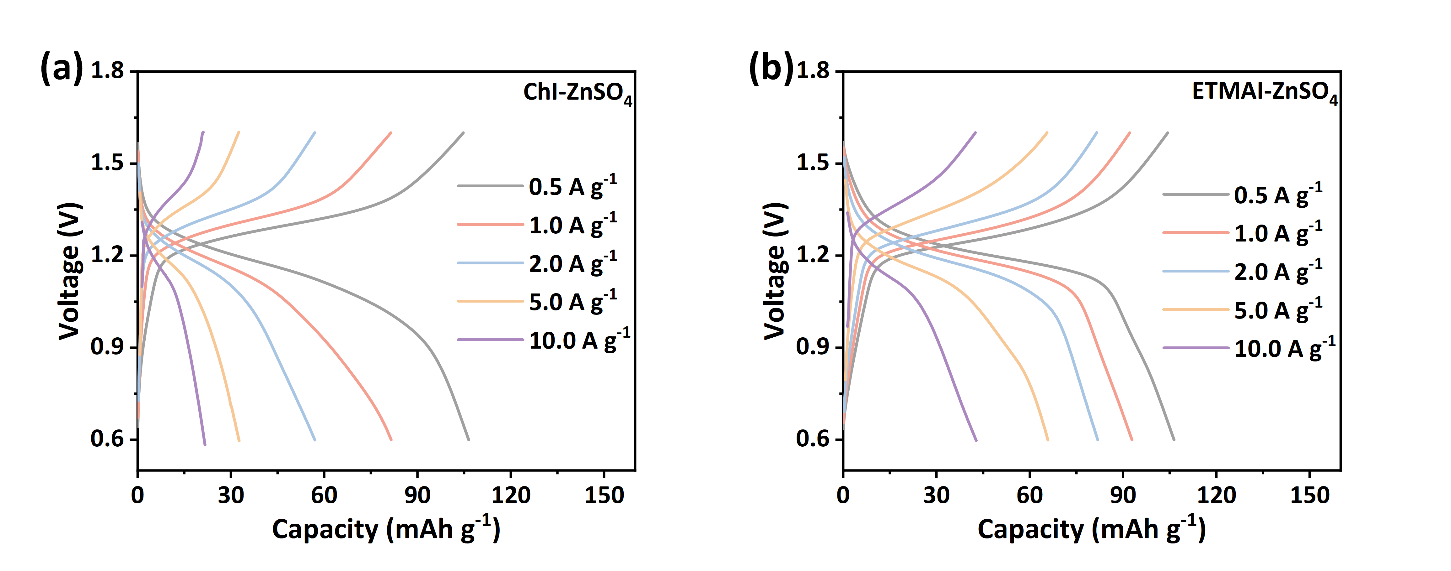


Figure S34. Charge-discharge curves of (a) ChI-ZnSO_4_ and (b) ETMAI-ZnSO_4_ electrolytes at various rates.


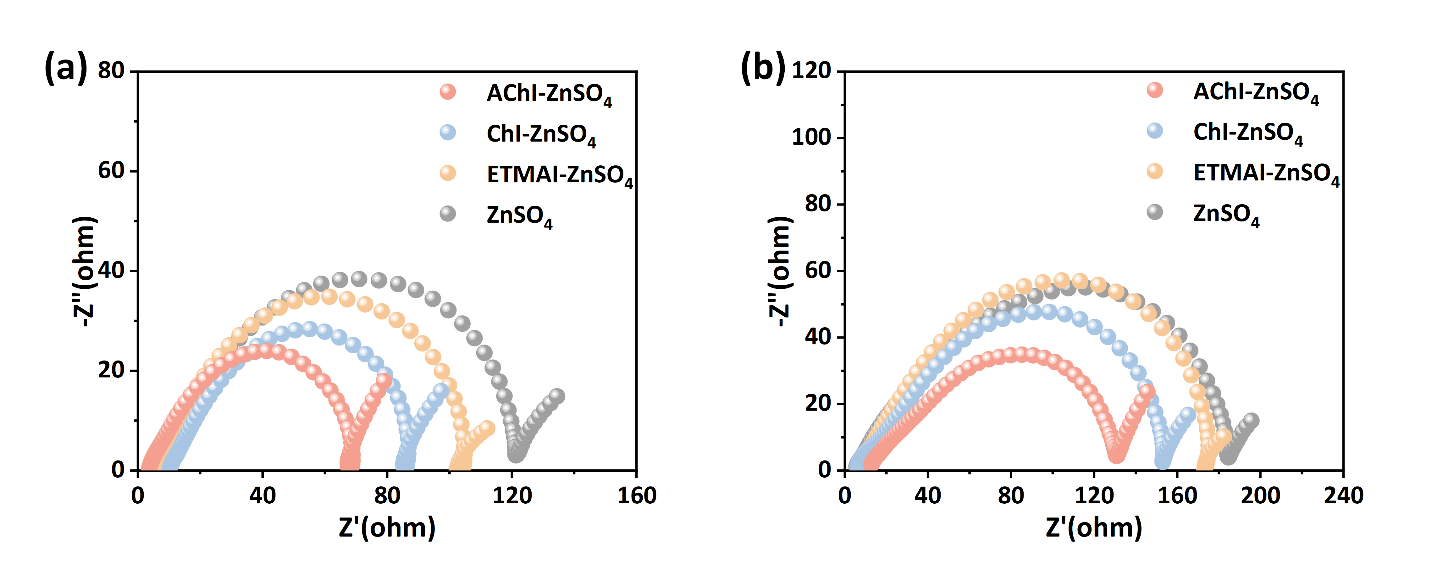


Figure S35. Nyquist plots of Zn//I_2_ full cells with different electrolytes before and after 500 cycles at 2 A g^-1^.


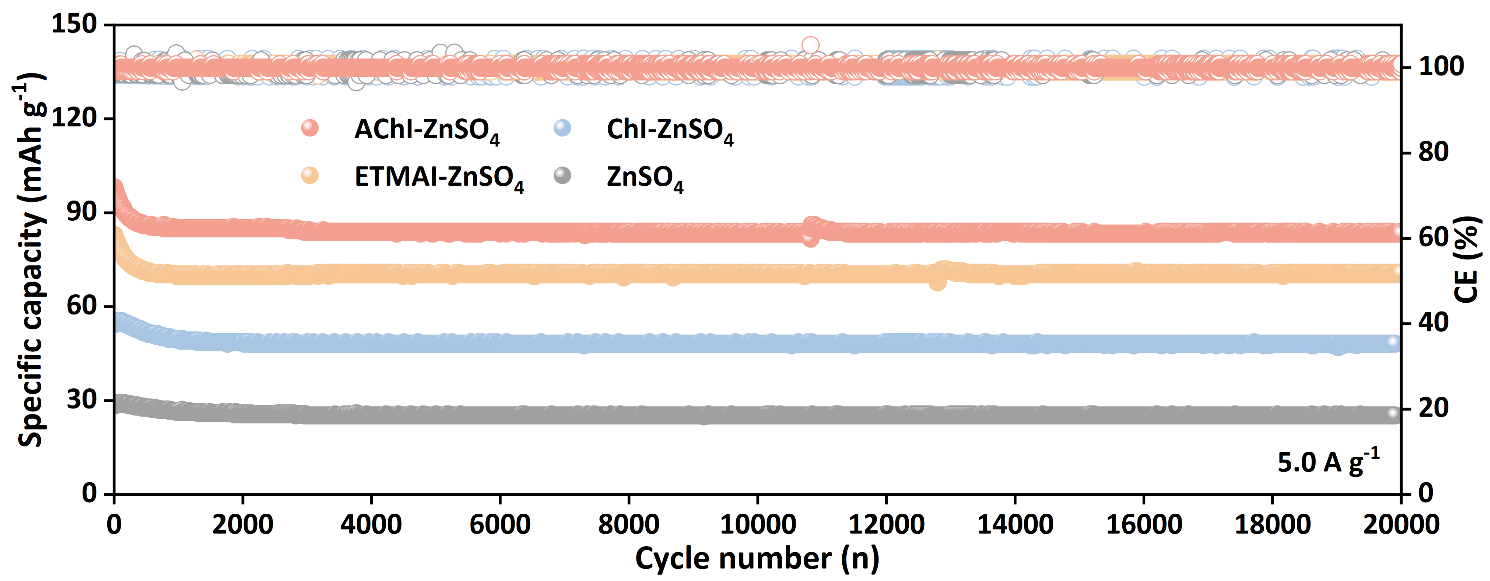


Figure S36. Long-term cycling performances of Zn//I_2_ full cells with different electrolytes at 5.0 A g^-1^.

Figure S37. Long-term cycling performance of the Zn-I_2_ battery in AChI-ZnSO_4_ electrolyte at 2.0 A g^-1^.( The iodine loading is 2.5 mg cm^-2^)


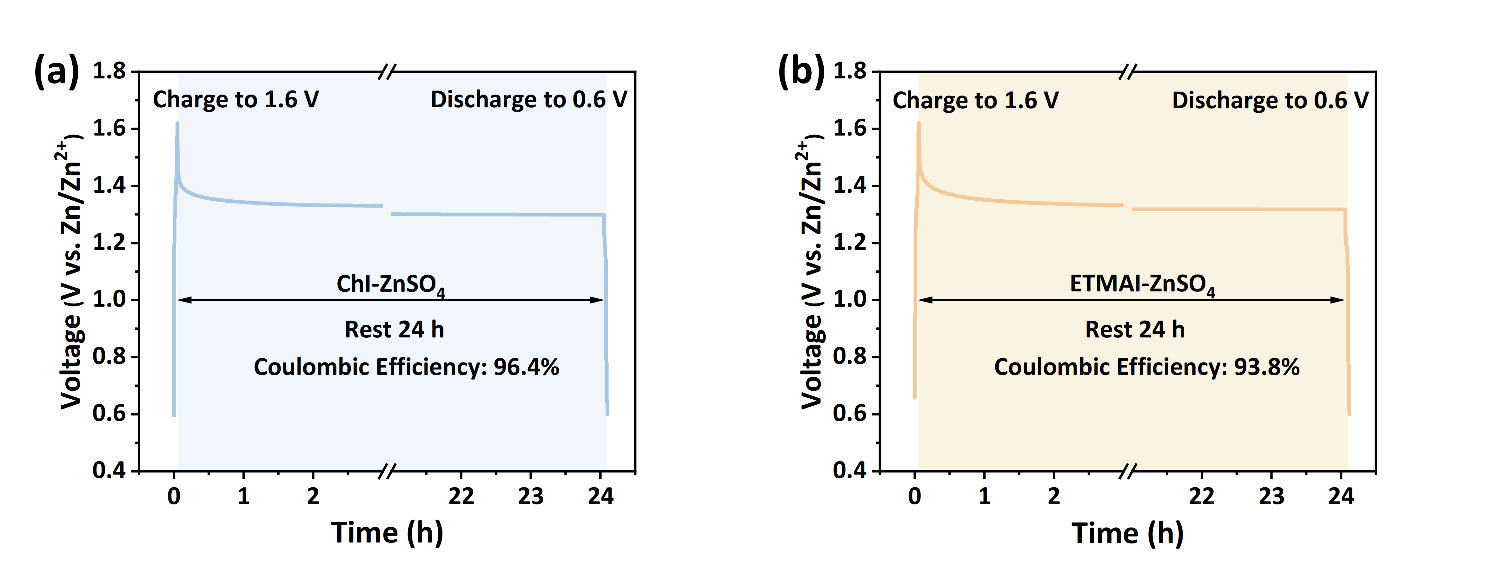


Figure S38. Self-discharge test of Zn//I_2_ full cells using (a) ChI-ZnSO_4_ and (b) ETMAI-ZnSO_4_ electrolytes.


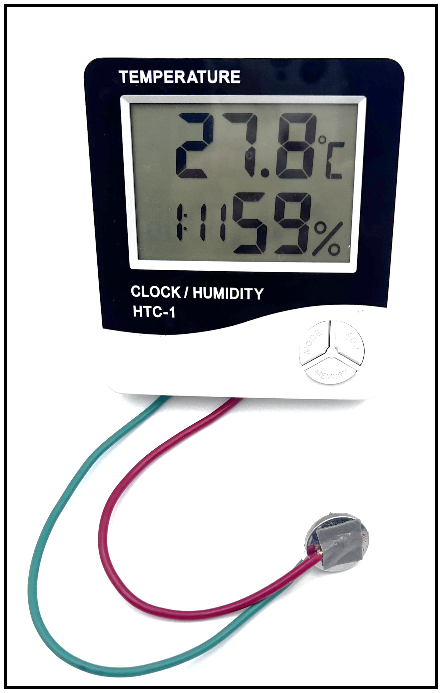


Figure S39. Digital photo of a hygrometer powered by Zn//I_2_ full cell with AChI-ZnSO_4_ electrolyte.

**Table S1.** Composition of simulation boxes.

|  | **Zn^2+^** | **SO_4_^2-^** | **H_2_O** | **ACh^+^/Ch^+^/ETMA^+^** | **I^-^** |
| --- | --- | --- | --- | --- | --- |
| 1 | 250 | 250 | 6944 | 0 | 0 |
| 2 | 250 | 250 | 6944 | 1 | 1 |

Table S2. Comparison of the cycling stability of Zn//Zn symmetric cells with recently reported electrolytes.

| **Electrolyte** | **Additive** | **Current density (mA cm^-2^)/**  **Capacity density (mAh cm^-2^)** | **Lifespan (h)** | **Reference** |
| --- | --- | --- | --- | --- |
| 2 M ZnSO_4_ | 8 mM AChI | **1-1** | **3700** | **This work** |
|  |  | **5-5** | **800** |  |
|  |  | **10-1** | **1500** |  |
| 2M ZnSO_4_ | 0.2 M SADS | 1-1 | 2100 | ^[12]^ |
|  |  | 5-5 | 500 |  |
| 2M ZnSO_4_ | 0.5 M CP | 1-1 | 1120 | ^[13]^ |
|  |  | 10-5 | 800 |  |
| 2 M ZnSO_4_ | 200 mM honey | 1-1 | 2000 | ^[14]^ |
| 2 M ZnSO_4_ | 0.4 m PCA-Zn | 1-1 | 1200 | ^[15]^ |
| 2 M ZnSO_4_ | 30% FA | 1-0.5 | 1600 | ^[16]^ |
| 2 M ZnSO_4_ | 0.1 g L^-1^ SDS | 1-1 | 2000 | ^[17]^ |
|  |  | 5-2.5 | 500 |  |
| 2 M ZnSO_4_ | 1 g L^-1^ SDBS | 0.2-0.2 | 1035 | ^[18]^ |
|  |  | 0.5-0.5 | 600 |  |
| 2 M ZnSO_4_ | 0.3 M Gln | 1-1 | 700 | ^[19]^ |
|  |  | 3-3 | 700 |  |
| 2 M ZnSO_4_ | 5 mM Na_3_NTA | 1-1 | 3000 | ^[20]^ |
|  |  | 5-2.5 | 700 |  |
| 2 M ZnSO_4_ | 50 mM TXA | 1-1 | 2100 | ^[21]^ |
|  |  | 2-2 | 500 |  |
|  |  | 5-5 | 700 |  |
| 2 M ZnSO_4_ | 1 mM NH_3_·H_2_O | 1-1 | 1500 | ^[22]^ |
|  |  | 3-3 | 450 |  |
|  |  | 5-5 | 250 |  |
| 2 M ZnSO_4_ | 15 mg ml^-1^ CMC | 1-0.5 | 3600 | ^[23]^ |
|  |  | 20-1 | 1350 |  |
| 2 M ZnSO_4_ | 0.5M Gly | 1-1 | 3200 | ^[24]^ |
|  |  | 10-10 | 460 |  |
|  |  | 20-20 | 80 |  |
| 2M ZnSO_4_ | 10 mg L^-1^ NSQDs | 2-0.5 | 1300 | ^[25]^ |
|  |  | 20-1 | 600 |  |
| 1 M ZnSO_4_ | 10 mM Suc | 2-2 | 500 | ^[26]^ |
|  |  | 5-5 | 300 |  |
| 1 M ZnSO_4_ | 0.5 M SBT | 1-1 | 1000 | ^[27]^ |
|  |  | 5-5 | 480 |  |

Table S3. Comparison of the Coulombic efficiency (CE) of recently reported electrolytes.

| **Electrolyte** | **Additive** | **Current density (mA cm^-2^)/**  **Capacity density (mAh cm^-2^)** | **Lifespan (h)** | **CE (%)** | **Reference** |
| --- | --- | --- | --- | --- | --- |
| 2 M ZnSO_4_ | 8 mM AChI | **1-0.5** | **2200** | **99.82** | **This work** |
|  |  | **2-0.5** | **2000** | **99.68** |  |
|  |  | **5-1** | **400** | **99.36** |  |
| 2M ZnSO_4_ | 0.2 M SADS | 2-1 | 200 | 99.2 | ^[12]^ |
| 2M ZnSO_4_ | 0.5M CP | 5-2.5 | 500 | 99.5 | ^[13]^ |
| 2 M ZnSO_4_ | 200 mM honey | 1-1 | 1000 | 99.8 | ^[14]^ |
| 2 M ZnSO_4_ | 0.4 m PCA-Zn | 1-1 | 700 | 99.43 | ^[15]^ |
| 2 M ZnSO_4_ | 30% FA | 1-0.5 | 1600 | 99.24 | ^[16]^ |
| 2 M ZnSO_4_ | 0.1 g L^-1^ SDS | 1-0.5 | 2000 | 99.42 | ^[17]^ |
| 2 M ZnSO_4_ | 1 g L^-1^ SDBS | 1-1 | 150 | / | ^[18]^ |
| 2 M ZnSO_4_ | 0.3 M Gln | 1-0.5 | 500 | 99.6 | ^[19]^ |
| 2 M ZnSO_4_ | 5 mM Na_3_NTA | 1-0.5 | 600 | 99.75 | ^[20]^ |
| 2 M ZnSO_4_ | 50 mM TXA | 1-0.5 | 1000 | 99.6 | ^[21]^ |
| 2 M ZnSO_4_ | 1 mM NH_3_·H_2_O | 2-1 | 100 | / | ^[22]^ |
| 2 M ZnSO_4_ | 15 mg ml^-1^ CMC | 1-0.5 | 1000 | 98.8 | ^[23]^ |
| 2 M ZnSO_4_ | 0.5M Gly | 2-2 | 650 | 99.68 | ^[24]^ |
| 2M ZnSO_4_ | 10 mg L^-1^ NSQDs | 5-1 | 600 | 99.8 | ^[25]^ |
| 1 M ZnSO_4_ | 10 mM Suc | 1-0.5 | 700 | 98.6 | ^[26]^ |
| 1 M ZnSO_4_ | 0.5 M SBT | 0.5-0.5 | 100 | 96.9 | ^[27]^ |

Table S4. Cycling performance of recently reported Zn-I_2_ batteries.

| **Material(s)** | **Current density**  **(A·g^-1^)** | **Cycle number** | **Capacity retention rates (%)** | **Specific capacity (mAh g^-1^)** | **Capacity decay per cycle (%)** | **Reference** |
| --- | --- | --- | --- | --- | --- | --- |
| 2 M ZnSO_4_-8 mM AChI | 2 | **25000** | **87.2** | **115.3** | **5.12*10^-4^** | **This work** |
|  | 5 | **20000** | **85.0** | **86.4** | **7.5*10^-4^** |  |
| Zn@Sn-ZnF_2_ | 2 | 20000 | 80 | 104 | 1*10^-3^ | ^[28]^ |
| Alginate-based hydrogel electrolyte | 1 | 2000 | 66.8 | 75.3 | 1.66*10^-2^ | ^[29]^ |
| ZnSO_4_-pyridine | 2 | 10000 | 92 | 135 | 8*10^-4^ | ^[30]^ |
| Zn-BTC membrane | 0.16 | 100 | 99 | 135 | 1*10^-2^ | ^[31]^ |
| ZnCl_2_ + KCl | 3 | 2800 | 80 | / | 7.14*10^-3^ | ^[32]^ |
| ODA host | 4.5 | 10000 | 80 | / | 2*10^-3^ | ^[33]^ |
| SC-PPS@Zn | 3.2 | 6000 | 90.2 | 84.6 | 1.63*10^-3^ | ^[34]^ |
| MPC/I_2_+KB@CF | 0.1 | 300 | 96.4 | 137 | 1.2*10^-2^ | ^[35]^ |
|  | 1 | 2000 | 40 | 112 | 3*10^-2^ |  |
| With Starch  cathodes | 2 | 10000 | / | 90.2 | / | ^[36]^ |
| 30 m ZnCl_2_ | 2 | 2000 | 95.7 | / | 5.22*10^-4^ | ^[37]^ |

**References**

[1] M. J. Abraham, T. Murtola, R. Schulz, S. Páll, J. C. Smith, B. Hess, E. Lindahl, *SoftwareX* **2015**, *1-2*, 19-25.

[2] A. W. Sousa da Silva, W. F. Vranken, *BMC Research Notes* **2012**, *5*, 367.

[3] C. I. Bayly, P. Cieplak, W. Cornell, P. A. Kollman, *The Journal of Physical Chemistry* **1993**, *97*, 10269-10280.

[4] J. Wang, W. Wang, P. A. Kollman, D. A. Case, *J Mol Graph Model* **2006**, *25*, 247-260.

[5] a)L. Su, F. Lu, J. Dong, X. Dou, L. Zheng, C. Ouyang, X. Gao, *Advanced Energy Materials* **2024**, *14*, 2400548; b)Y. Zhang, G. Wan, N. H. C. Lewis, J. Mars, S. E. Bone, H.-G. Steinrück, M. R. Lukatskaya, N. J. Weadock, M. Bajdich, O. Borodin, A. Tokmakoff, M. F. Toney, E. J. Maginn, *ACS Energy Letters* **2021**, *6*, 3458-3463; c)E. Duboué-Dijon, P. Delcroix, H. Martinez-Seara, J. Hladílková, P. Coufal, T. Krizek, P. Jungwirth, *The Journal of Physical Chemistry B* **2018**, *122*.

[6] S. Nosé, M. L. Klein, *Physical Review Letters* **1983**, *50*, 1207-1210.

[7] E. F. Pettersen, T. D. Goddard, C. C. Huang, E. C. Meng, G. S. Couch, T. I. Croll, J. H. Morris, T. E. Ferrin, *Protein Sci* **2021**, *30*, 70-82.

[8] D. M. York, T. A. Darden, L. G. Pedersen, *The Journal of Chemical Physics* **1993**, *99*, 8345-8348.

[9] W. Humphrey, A. Dalke, K. Schulten, *J Mol Graph* **1996**, *14*, 33-38, 27-38.

[10] a)Y. Wang, J. Lv, L. Hong, J. Zhang, C. Chen, A. Xu, M. Huang, X. Ren, J. Bai, H. Wang, X. Liu, *Angewandte Chemie International Edition* **2024**, *64*, e202414757; b)S. Qin, Y. Liu, R. Li, Y. Jiao, H. Chen, J. Zhao, *Journal of Materials Chemistry A* **2024**, *12*, 5805-5814.

[11] L. Su, F. Lu, Y. Li, Y. Wang, X. Li, L. Zheng, X. Gao, *ACS Nano* **2024**, *18*, 7633-7643.

[12] Y. Ding, L. Yin, T. Du, Y. Wang, Z. He, J. A. Yuwono, G. Li, J. Liu, S. Zhang, T. Yang, Z. Guo, *Advanced Functional Materials* **2024**, *34*, 2314388.

[13] Q. Yan, Z. Hu, Z. Liu, F. Wu, Y. Zhao, R. Chen, L. Li, *Energy Storage Materials* **2024**, *67*, 103299.

[14] F. Tan, X. Cai, W. Yan, Q. Wang, J. Zhao, W. Zhang, *Energy Storage Materials* **2024**, *67*, 103273.

[15] M. Yang, J. Zhu, S. Bi, R. Wang, H. Wang, F. Yue, Z. Niu, *Angewandte Chemie International Edition* **2024**, *63*, e202400337.

[16] X. Li, J. Miao, F. Hu, K. Yan, L. Song, H. Fan, L. Ma, W. Wang, *Journal of Materials Chemistry A* **2024**, *12*, 968-978.

[17] F. Jing, L. Xu, Y. Shang, G. Chen, C. Lv, C. Yan, *Journal of Colloid and Interface Science* **2024**, *669*, 984-991.

[18] X. Yang, Q. Zhou, S. Wei, X. Guo, P. J. Chimtali, W. Xu, S. Chen, Y. Cao, P. Zhang, K. Zhu, H. Shou, Y. Wang, X. Wu, C. Wang, L. Song, *Small Methods* **2024**, *8*, 2301115.

[19] J. Yin, M. Li, X. Feng, T. Cui, J. Chen, F. Li, M. Wang, Y. Cheng, S. Ding, X. Xu, J. Wang, *Journal of Materials Chemistry A* **2024**, *12*, 1543-1550.

[20] Z. Jiao, X. Cai, X. Wang, Y. Li, Z. Bie, W. Song, *Advanced Energy Materials* **2023**, *13*, 2302676.

[21] J. Yin, H. Liu, P. Li, X. Feng, M. Wang, C. Huang, M. Li, Y. Su, B. Xiao, Y. Cheng, X. Xu, *Energy Storage Materials* **2023**, *59*, 102800.

[22] R. Chen, W. Zhang, Q. Huang, C. Guan, W. Zong, Y. Dai, Z. Du, Z. Zhang, J. Li, F. Guo, X. Gao, H. Dong, J. Zhu, X. Wang, G. He, *Nano-Micro Letters* **2023**, *15*, 81.

[23] H. Huang, J. Yun, H. Feng, T. Tian, J. Xu, D. Li, X. Xia, Z. Yang, W. Zhang, *Energy Storage Materials* **2023**, *55*, 857-866.

[24] Y. Liu, Y. An, L. Wu, J. Sun, F. Xiong, H. Tang, S. Chen, Y. Guo, L. Zhang, Q. An, L. Mai, *ACS Nano* **2023**, *17*, 552-560.

[25] F. Wang, H. Lu, H. Zhu, L. Wang, Z. Chen, C. Yang, Q.-H. Yang, *Energy Storage Materials* **2023**, *58*, 215-221.

[26] C. Wang, J. Hou, Y. Gan, L. Xie, Y. He, Q. Hu, S. Liu, S. Chan Jun, *Journal of Materials Chemistry A* **2023**, *11*, 8057-8065.

[27] M. Qiu, P. Sun, A. Qin, G. Cui, W. Mai, *Energy Storage Materials* **2022**, *49*, 463-470.

[28] G. Wang, Q. Yao, J. Dong, W. Ge, N. Wang, Z. Bai, J. Yang, S. Dou, *Advanced Energy Materials* **2024**, *14*, 2303221.

[29] W. Shang, J. Zhu, Y. Liu, L. Kang, S. Liu, B. Huang, J. Song, X. Li, F. Jiang, W. Du, Y. Gao, H. Luo, *ACS Applied Materials & Interfaces* **2021**, *13*, 24756-24764.

[30] Y. Lyu, J. A. Yuwono, P. Wang, Y. Wang, F. Yang, S. Liu, S. Zhang, B. Wang, K. Davey, J. Mao, Z. Guo, *Angewandte Chemie International Edition* **2023**, *62*, e202303011.

[31] H. Yang, Y. Qiao, Z. Chang, H. Deng, P. He, H. Zhou, *Advanced Materials* **2020**, *32*, 2004240.

[32] X. Li, M. Li, Z. Huang, G. Liang, Z. Chen, Q. Yang, Q. Huang, C. Zhi, *Energy & Environmental Science* **2021**, *14*, 407-413.

[33] X. Li, S. Wang, T. Wang, Z. Duan, Z. Huang, G. Liang, J. Fan, C. Yang, A. L. Rogach, C. Zhi, *Nano Energy* **2022**, *98*, 107278.

[34] L. Zhang, J. Huang, H. Guo, L. Ge, Z. Tian, M. Zhang, J. Wang, G. He, T. Liu, J. Hofkens, D. J. L. Brett, F. Lai, *Advanced Energy Materials* **2023**, *13*, 2203790.

[35] Y. Hou, F. Kong, Z. Wang, M. Ren, C. Qiao, W. Liu, J. Yao, C. Zhang, H. Zhao, *Journal of Colloid and Interface Science* **2023**, *629*, 279-287.

[36] S.-J. Zhang, J. Hao, H. Li, P.-F. Zhang, Z.-W. Yin, Y.-Y. Li, B. Zhang, Z. Lin, S.-Z. Qiao, *Advanced Materials* **2022**, *34*, 2201716.

[37] G. Liang, B. Liang, A. Chen, J. Zhu, Q. Li, Z. Huang, X. Li, Y. Wang, X. Wang, B. Xiong, X. Jin, S. Bai, J. Fan, C. Zhi, *Nature Communications* **2023**, *14*, 1856.
